# A systemically administered detoxified TLR4 agonist displays potent antitumor activity and an acceptable tolerance profile in preclinical models

**DOI:** 10.3389/fimmu.2023.1066402

**Published:** 2023-05-08

**Authors:** Kamel Chettab, Chantel Fitzsimmons, Alexey Novikov, Morgane Denis, Capucine Phelip, Doriane Mathé, Pierre Antoine Choffour, Sabine Beaumel, Eric Fourmaux, Patrick Norca, David Kryza, Anne Evesque, Lars Petter Jordheim, Emeline Perrial, Eva-Laure Matera, Martine Caroff, Jerome Kerzerho, Charles Dumontet

**Affiliations:** ^1^ INSERM U1052, CNRS UMR 5286, Centre de Recherche en Cancérologie de Lyon, Université de Lyon, Lyon, France; ^2^ Hospices Civils de Lyon, Lyon, France; ^3^ HEPHAISTOS-Pharma, Université Paris-Saclay, Orsay, France; ^4^ Antinéo, Lyon, France

**Keywords:** modified TLR4 agonist, liposomal formulation, tolerance, *in vivo* antitumor activity, adjuvant effect, lipopolysaccharides

## Abstract

Bacterial lipopolysaccharides (LPS) are potent innate immunostimulants targeting the Toll-like receptor 4 (TLR4), an attractive and validated target for immunostimulation in cancer therapy. Although LPS possess anti-tumor activity, toxicity issues prevent their systemic administration at effective doses in humans. We first demonstrated that LPS formulated in liposomes preserved a potent antitumor activity *per se* upon systemic administration in syngeneic models, and significantly enhance the antitumor activity of the anti-CD20 antibody rituximab in mice xenografted with the human RL lymphoma model. Liposomal encapsulation also allowed a 2-fold reduction in the induction of pro-inflammatory cytokines by LPS. Mice receiving an intravenous administration demonstrated a significant increase of neutrophils, monocytes and macrophages at the tumor site as well as an increase of macrophages in spleen. Further, we chemically detoxified LPS to obtain MP-LPS that was associated with a 200-fold decrease in the induction of proinflammatory cytokines. When encapsulated in a clinically approved liposomal formulation, toxicity, notably pyrogenicity (10-fold), was limited while the antitumor activity and immunoadjuvant effect were maintained. This improved tolerance profile of liposomal MP-LPS was associated with the preferential activation of the TLR4-TRIF pathway. Finally, *in vitro* studies demonstrated that stimulation with encapsulated MP-LPS reversed the polarization of M2 macrophages towards an M1 phenotype, and a phase 1 trial in healthy dogs validated its tolerance upon systemic administration up to very high doses (10µg/kg). Altogether, our results demonstrate the strong therapeutic potential of MPLPS formulated in liposomes as a systemically active anticancer agent, supporting its evaluation in patients with cancer.

## 1 Introduction

Immunotherapy has emerged as a major player in the treatment of neoplasia. Most current immunomodulatory approaches focus on the controlled activation of the adaptive immune system, either by targeting inhibitory pathways with immune checkpoint inhibitors (ICIs), or by targeting activating pathways, as with genetically modified autologous T cells or bispecific antibodies (1). Although these therapies have led to unprecedented successes, only a minority of patients with cancer benefits from these treatments, highlighting the need to identify new cells and molecules that could be exploited in the next generation of immunotherapy. Innate immune cells are known to play a key role in the development of effective and long-lasting T-cell responses through antigen processing and presentation, production of key cytokines and to behave as anti-tumor effector cells (2). Their role in the control of cancer progression and in cancer therapy is also well documented (2). Given the crucial role of innate immune responses in immunity, harnessing these responses could open up new possibilities for more effective and sustainable tumour control.

LPS are well-known as powerful innate immunostimulants. They are glycolipids, mainly found in the outer membrane of Gram-negative bacteria, and composed of three distinct parts including the O-antigen, a core oligosaccharide, and a lipid A which is most frequently made of a phosphorylated glucosamine disaccharide substituted with different fatty acids (3, 4). LPS specifically binds to the TLR4 receptor expressed on professional antigen-presenting cells (APCs), such as dendritic cells (DC), monocytes, macrophages, and activated B cells, but also expressed at a lower level on some non-immune cells, including epithelium, endothelium, placental cells and beta cells in Langerhans islets (5). Binding of LPS to TLR4 involves the co-receptor MD2 (myeloid differentiation 2) and in the case of CD14+ cells, such as macrophages, it also involves the complexation with circulating LPS-binding protein (LBP) allowing the transfer of LPS to CD14 which facilitates its interaction with MD2 (6). TLR4 activation by LPS leads to the activation of two different signaling pathways which is unique in the TLR family (7). This includes the canonical pathway, mediated by MyD88 and NFκB leading to the production of pro-inflammatory cytokines, and the alternative MyD88 independent pathway involving the recruitment of TRIF (TIR-domain-containing adaptor containing interferon beta) and TRAM (TRIF-related adaptor molecule) and the activation of IRF3 (Interferon Regulatory Factor 3) resulting in the production of type 1 interferons (8). Accordingly, stimulation of TLR4 by LPS on APCs induces the secretion of both pro-inflammatory and type I interferons cytokines, and chemokines but also the stimulation of antigen presentation and upregulation of costimulatory molecules such as CD40 and CD80 (9, 10).

TLR4 engagement by LPS enables the onset, the recruitment, the polarization and the maintenance of effective and long-lasting T-cell responses (7, 10). Since its isolation in 1943, LPS have indeed demonstrated potent anti-tumor efficacy as systemic therapeutic agents in several tumor models (11, 12). However, clinical trials evaluating the intravenous administration of LPS to patients with cancer have reported limiting toxicities at doses in the ng/kg range, which are too low to obtain antitumor effects. These were defined by liver limiting toxicity associated with hematological changes and the release of pro-inflammatory cytokines, such as TNFα and IL-6, as well as macrophage colony-stimulating factor (M-CSF) (13).

Various strategies have been developed to improve LPS tolerance. The formulation of LPS in liposomes, but also the use of alternative routes of administration were found to improve LPS tolerance (10, 14–16). Among these, specific structural modifications are considered as the most efficient aproaches allowing dissociation of beneficial and deleterious properties of LPS molecules. LPS extracted from different bacterial strains display both molecular variations and differences in immunostimulatory properties. We have previously shown that *B. pertussis* LPS is less inflammatory than *Salmonella*-type LPS. This is due to natural hypo-acylation (5 fatty acids instead of 6 or 7 in Enterobacterial LPS, and presence of a short-chain 10:0(3OH), while Salmonella lipid A structures display at least four 14:0(3-OH) and two branched fatty acids. In the early 80’s, we demonstrated that the chemical removal of a phosphate group from the lipid A moiety of *Bordetella pertussis* leads to a non-toxic, non-pyrogenic molecule maintaining its immunostimulatory properties (17–19).

At the same period chemically dephosphorylated and partially de-O-acylated lipid A was obtained from *Salmonella minnesota* R595 LPS. This so-called detoxified Monophosphoryl lipid A (MPL) was notably approved as an adjuvant for human vaccines (20, 21). Later, some natural and synthetic LPS derivatives based on dephosphorylated lipid A moiety structures were also developed and confirmed potent adjuvant and antitumor activities (10, 22, 23). MPL developed by GSK is a natural mono-phosphoryl molecule with 2 to 6 acyl molecular species while the GLA from Merck is a synthetic MPL with a unique hexa-acyl molecular species. Both MPL and GLA are more lipophilic than native LPS since they are limited to a lipid A-like moiety without core sugars. Therefore, they aggregate in aqueous solutions and are not suitable for IV injection. Based on our study demonstrating that the lipid A moiety alone was less active than the full LPS molecule (24), we recently produced an innovative chemically-detoxified monophosphorylated LPS (MP-LPS) conserving a more complete structure of the native molecule than MPL since it contains both the core oligosaccharide and the lipid A moiety. MP-LPS displays a better tolerance than current LPS derivatives upon intravenous administration, while conserving potent antitumor and adjuvant effects.

Recently, the intratumoral administration of Glucopyranosyl Lipid Adjuvant (GLA-SE/G100), a synthetic detoxified analog of lipid A formulated in a stable emulsion, showed anti-tumor immune responses and tumor regression in patients with Merkel cell carcinoma, and adjuvant activity in combination with pembrolizumab in patients with follicular lymphoma (25–27). Although intratumoral injection of synthetic LPS derivatives has shown promising clinical results, abscopal effects, i.e. responses outside of the tumour sites injected with this compound, remained limited, impairing their efficacy in case of disseminated disease (27). Systemic administration could overcome these limitations but current LPS derivatives remain too toxic to be administered intravenously at efficient doses, as demonstrated by the recent Phase 1 clinical trials with GSK1795091, another synthetic LPS derivative (28). The development of a systemically active TLR4 agonist thus requires the identification of new less toxic molecules.

In the present study, we sought to examine the impact of liposomal formulations on the immunostimulatory, antitumor activities and tolerance profile of an unmodified commercial LPS and MP-LPS. Our results show that the chemical detoxification process and the liposomal formulation can act synergistically to produce a systemically active and well tolerated TLR4 agonist which retains potent antitumor and immunoadjuvant properties.

## 2 Material and Methods

### 2.1 Reagents

1,2-dioleoyl-*sn*-glycero-3-phosphoethanolamine (DOPE), 1,2-distearoyl-*sn*-glycero-3 phosphoethanolamine-N-[methoxy(polyethylene glycol)-5000] (18:0 PEG5000 PE), 1,2-distearoyl-*sn*-glycero-3-phosphoethanolamine-N-[methoxy(polyethylene glycol)-350] (18:0 PEG350 PE), 1,2-dioleoyl-sn-glycero-3-phosphoethanolamine-N-(lissamine rhodamine B sulfonyl) [DOPE-Rh B), 1,2-dimyristoyl-sn-glycero-3-phosphocholine (DMPC), 1,2-dimyristoyl-sn-glycero-3-phospho-(1’-rac-glycerol) (DMPG), 1,2-dioleoyl-sn-glycero-3-phosphoethanolamine-N-(7-nitro-2-1,3-benzoxadiazol-4-yl) (ammonium salt) (18:1 NBD PE) and 1-myristoyl-2-{6-[(7-nitro-2-1,3-benzoxadiazol-4-yl) amino]hexanoyl}-sn-glycero-3-[phospho-rac-(1-glycerol)] (ammonium salt) (14:0-06:0 NBD PG) were obtained from Avanti Polar Lipids (Alabaster, AL, USA). Phorbol 12-myristate 13-acetate (PMA), cholesterol and Lipopolysaccharides (LPS) from *E. coli*, serotype O55:B5 were purchased from Sigma-Aldrich. Rituximab (MabThera, Roche) a chimeric monoclonal antibody directed against CD20 was purchased as its commercial formulation and obinutuzumab (GA101) a glycoengineered Type II CD20 monoclonal antibody was kindly provided by Roche. Anti-PD-1 immune checkpoint inhibitor RMP1-14 was from BioXCell.

### 2.2 Chemically detoxified LPS

The chemically detoxified LPS, Monophosphoryl-LPS (MP-LPS), was produced and provided by the company HEPHAISTOS-Pharma (Orsay, France). Briefly, LPS from *Bordetella pertussis* was extracted from a culture pellet as previously described (29). The extracted LPS were then chemically modified using an innovative process including mild alkaline and acidic treatments, enabling to remove a phosphate group from the lipid A moiety without consecutive cleavage of the acido-labile Kdo linkage of the core oligosaccharide. Structural quality control analyses were performed on MP-LPS by matrix assisted laser desorption mass spectrometry (MALDI-MS - Shimadzu AXIMA Performance time-of-flight mass spectrometer, in linear mode with delayed extraction) and SDS-polyacrylamide gel electrophoresis (SDS-PAGE) as previously described (29). The level of supramolecular aggregate formation, as well as solubility, both in water, were assessed by Dynamic light scattering (DLS). Chemical purity, including amino and nucleic acids contents, was evaluated respectively by LC-MS (Hitachi L-8800 amino acids analyser with a 2620MSC-PS column) and UV spectrophotometry (absorbance at 260 nm - Denovix spectrophotometer). Immunological purity was also controlled by evaluating the level of activation of the TLR4 and TLR2 pathways using *in vitro* assays on HEK-Blue TM hTLR4 and hTLR2 transfected reporter cells (Invivogen).

### 2.3 Cell lines and culture

RL (ATCC: CRL-226), a human NHL B-cell line expressing CD20 and A20, a BALB/c B-cell lymphoma line (ATCC-TIB-208) were cultured in RPMI1640 media, 10% FCS, 100 U/mL of penicillin and 100 μg/mL of streptomycin (all media reagents from Life Technologies) at 37°C in a 5% CO2 atmosphere. The K7M2 murine osteosarcoma cell line (*ATCC CRL-2836*, *ATCC*) and murine colon carcinoma cell line MC38 (KeraFAST, Boston, MA, USA) were cultured in Dulbecco’s modified Eagle’s medium (DMEM) supplemented with 10% fetal bovine serum, 100 U/mL penicillin/streptomycin. Cell culture was carried out in accordance with good laboratory practices including genotyping of cell lines used in this study. The expression of cell surface markers CD20 was assessed by flow cytometry on RL cells. Using MycoAlert Kit (Lonza) all cells used in this study were regularly tested mycoplasma negative.

### 2.4 Liposome preparation and characterization

Liposomes were prepared by the conventional thin film hydration procedure. The compositions of liposome formulations 1 and 2 (F1Lipo and F2Lipo) were the following, respectively: DOPE: DSPE-PEG 5000: DSPE-PEG-350: Chol (54:8:8:30 mol %) and DMPC: DMPG (70:30 mol%). Briefly, the mixture was dissolved in chloroform: methanol (9:1 v/v) in round-bottomed flask, vacuum-desiccated using a rotary evaporator and hydrated with sterilized phosphate-buffered saline, pH 7.4 (PBS) for liposomes, and with LPS solution (100 μg/mL) in sterilized PBS for Lipo LPS. The liposome preparation was frozen and thawed for three cycles. A single freeze-thaw cycle consisted of freezing for 15 min at liquid nitrogen temperature and thawing for 15 min in a water bath at 25°C. The liposome formulations were then downsized by stepwise extrusion (Lipex extruder, Biomembrane Inc., Vancouver B.C Canada) through Nucleopore polycarbonate filters with decreasing pore size from 800 to 200 nm (Nuclepore, West Chester, PA). The hydrodynamic diameter and polydispersity (PDI) of the Lipo LPS preparation was measured by dynamic light scattering (DLS) using a Zetasizer Nano-S from Malvern instrument (Worcestershire, UK). Finally, the zeta-potential of the liposome preparation was measured using a Zetasizer Nano-Zs (Malvern, Worcestershire, UK). To study the distribution of liposomes *in vivo*, these were radiolabeled on the surface with ^111^In using 18:0 PE: DTPA a chelating lipid inserted in the bilayer. The radiolabeled liposome formulation was thus: DOPE: PEG5000: PEG350: cholesterol: PE-DTPA (54:7.5: 8: 30: 0.5 mol%).

### 2.5 Microscopic analyses of Lipo LPS

Evaluation of the distribution of LPS within the liposomes was performed by confocal microscopy. The F1liposome bilayer was labeled with 0.5 mol % of DOPE-Rhodamine B and fluorescent FITC-LPS was encapsulated. The resulting liposomal formulation was examined using a Leica TCS SP5 confocal microscope (Leica Microsystems, Germany). Evaluation of morphology and architecture of liposomes was performed by transmission electron microscopy (TEM) using the negative staining method (CIQLE platform, University of Lyon).

### 2.6 Encapsulation efficiency

LPS encapsulation efficiency (EE) of the different LPS was tested by estimation of their respective characteristic 3-OH fatty acid content by LC-MS^2^ analysis as these fatty acids, not being present in other natural molecules, are known as good LPS markers. After centrifugation at 150 000g (1h at 4°C), the fatty acid amount was estimated in both pellets and supernatants. The fatty acid amount in the pellets accounted for the incorporated LPS molecules and the fatty acid amount in the supernatants accounted for the free LPS molecules, not incorporated in liposomes. Results were expressed in percentage of incorporation by comparison to the fatty acid content of each LPS molecule considered.

### 2.7 Monocyte isolation and activation test

Blood samples from healthy donors were provided by the Lyon Blood Bank. Peripheral Blood Mononuclear Cells (PBMC) were obtained by performing a density gradient centrifugation (Pancol, Pan-Biotech). Remaining red blood cells were removed using a lysis solution (BD Pharm Lyse, BD Biosciences) and cells were maintained in culture at 37°C with 5% CO2 in RPMI medium supplemented with 10% heat inactivated fetal calf serum (FCS), 200 UI/mL of penicillin and 200 μg/mL of streptomycin. After one-hour, non-adherent cells were harvested by thorough washing with PBS. Adherent cells were stimulated with unformulated LPS or Lipo LPS for 1 ½ hours, at various concentrations.

### 2.8 *In vitro* binding of liposomal formulations to fresh leukocytes

In order to analyze the liposome-leukocyte interaction, the liposome bilayer was labeled with 0.05 mol % of fluorophore NBD. FITC-labeled LPS was used to produce F1Lipo-LPS-FITC.

The resulting labeled liposomal preparations (emptyF1Lipo-NBD, F1Lipo-NBD-LPS, emptyF2Lipo-NBD and F2Lipo-NBD-MP-LPS) were incubated with fresh normal human leukocytes and F1Lipo-LPS-FITC was incubated with fresh normal mouse leukocytes. Liposome-leucocyte interactions were analyzed by flow cytometry. The Lyon Blood Bank provided blood samples from healthy donors, and murine leukocytes were obtained from BALB/C mice spleens. After blood collection in BD Vacutainers containing lithium heparin as an anticoagulant, 200 μl of normal human blood was distributed into 1.5 mL Eppendorf tubes containing liposome preparations (20 μL) and incubated for 3 h at 37°C with rotation. After incubation, red blood cells were removed using a lysis solution (BD Pharm Lyse, BD Biosciences). The remaining cells were incubated with human IgG for 10 min on ice to block unspecific binding and stained in 100 µL PBS for 45 min at 4°C with APC anti-human CD45 before being subjected to flow cytometry. The binding of leukocytes with DNB‐liposomes (Ex 467 nm/Em 539 nm) was monitored by flow cytometry. In the case of mice splenocytes, cells were incubated with liposome preparations for 3 hours. Cells were then stained in 100 µL PBS for 30 min at 4°C with a viability dye (65-0865-14, eBioscience), anti-CD45-V500 (561487, BD), anti-CD11b-BV605 (83-0112-42, Invitrogen), anti-CD4-BV650 (563747, BD), anti-CD8-PercP (553036, BD), anti-CD19-APC (550992, BD), anti-Ly6G-AF700 (561236, BD), anti-Ly6C-APC-Cy7 (560596, BD), anti-CD3-UV2 (564380, BD). Samples were acquired with a FortessaX20 flow cytometer (BD) with Diva software (BD). FlowJo-V10.7.1 software (BD) was used for analyses.

### 2.9 Real-time quantitative PCR

Total RNA was extracted using the Qiagen RNeasy Mini Kit (Cat. 74,106) according to the manufacturer’s instructions. RNA purity and concentration were assessed, and cDNA was synthesized using SuperScript IV Reverse Transcriptase (Thermo Fisher Scientific). Quantitative RT-PCR was performed on a Roche LightCycler^®^ 480 instrument using gene-specific primers and Takyon SYBR^®^ Master Mix (Eurogentec, Takyon No Rox SYBR^®^ MasterMix dTTP Blue). The primers used are listed in **Supplementary Table 1**. Reactions were performed in triplicate as follows: 95°C for 10 min, followed by 40 cycles of denaturation at 95°C for 10 s, annealing at 60°C for 10 s, and extension at 72°C for 15 s. Results were analyzed using the 2^−ΔΔ^ *Ct* method and normalized to the corresponding level of the housekeeping gene.

### 2.10 Antibody dependent cellular phagocytosis

Effect of LPS, F1Lipo-LPS, MP-LPS and F2Lipo-MP-LPS on the phagocytic activity of normal human leukocytes was assessed with the FagoFlow method according to the manufacturer’s instructions (Exbio Diagnostics) in order to measure the oxidative burst in granulocytes after stimulation. The Lyon Blood Bank provided blood samples from healthy donors. After blood collection in BD Vacutainers containing lithium heparin as an anticoagulant, 400 μL of normal human blood was distributed into 1.5 mL Eppendorf tubes containing the different molecules to be evaluated (LPS, F1Lipo-LPS, MP-LPS and F2Lipo-MP-LPS) in the presence of *Dihydrorhodamine 123* (DHR 123) and incubated for 1 h at 37°C with rotation. In the presence of ROS, nonfluorescent DHR123 is oxidized to fluorescent rhodamine 123 and is detected with FITC channel (525nm). After incubation, red blood cells were removed using a lysis solution (BD Pharm Lyse, BD Biosciences). The remaining cells were incubated with human IgG for 10 min on ice to block unspecific binding and stained in 100 µL PBS for 45 min at 4°C with APC-H7 anti-human CD45 before being subjected to flow cytometry. Gating was performed as shown in **Supplementary Figure 1**. Leukocyte activation was evaluated by measuring the phagocytic activity of normal human leukocytes, in an Antibody-Dependent Cellular Phagocytosis (ADCP) assay as previously described (30).

### 2.11 Radiolabeling of liposomes

In order to determine the *in vivo* localization of F1Lipo-LPS, LPS-containing liposomes were labeled with ^111^In. Briefly, LPS-containing DTPA-PE F1liposomes (200µL) were radiolabeled by adding 200 μl of acetate buffer 100 mM pH 5 and 40–100 MBq of high purity 111In-chloride (Covidien, Petten, Netherlands). The mixture was incubated for 30 minutes at 37°C. Free indium 111 was removed using a PD-10 column. The column was first washed with 15 mL of acetate buffer 0.1 M, then the labeled mixture was loaded on the column and eluted using acetate buffer 100mM. ^111^In-DTPA-PE encapsulated LPS were first eluted. Radiochemical purity (RCP) of each 0.5 mL fraction was evaluated using ITLC-SG (Biodex, Tec-control black) and citrate buffer 50 mM (pH 5) as mobile phase. Radiolabeled ^111^In-DTPA-PE encapsulated LPS remained at the origin whereas unbound ^111^In migrated with an Rf of 0.9–1. The highest radiochemical purity fractions were pooled. For stability testing, an aliquot of the radiolabeled ^111^In-DTPA-PE encapsulated LPS was incubated at 37°C in 2 mL phosphate buffer saline (pH 7.4) and RCP was evaluated using ITLC-SG and citrate buffer 0.1M pH5 as mobile phase.

### 2.12 Quantitative biodistribution and imaging

Eight to 15 MBq of radiolabeled ^111^In-DTPA-PE encapsulated LPS in a volume of 100 µL were intravenously injected into immunocompetent C57BL/6 mice bearing established subcutaneous colorectal MC38 tumors (n=3 for each group). Mice were sacrificed at 4h and 72h after injection by cervical dislocation. Tissues of interest (blood, heart, lungs, spleen, kidneys, muscles, brain, and skin) were removed, weighed and radioactivity was counted in a gamma scintillation counter (Wizard^®^ gamma counter, Perkin Elmer, USA). Urine and feces were collected thanks to individual metabolic cages for housing animals and counts. Tissue distribution was expressed as the percentage of injected dose per gram (%ID/g). Renal and hepatobiliary elimination were expressed as cumulated radioactivity over total injected activity. Imaging was performed using a small animal Nano-SPECT/CT system (Bioscan, Washington, DC, USA). SPECT/CT acquisitions were performed after IV injection of 8-15 MBq of radiolabeled ^111^In-DTPA-PE encapsulated LPS at 4h and 72h. X-ray CT (tube voltage of 55 kVp, exposure time of 500 ms, and 180 projections) and SPECT acquisitions were performed in mice bearing tumors in a supine position, placed in a temperature-controlled bed (Minerve, Esternay, France), in order to maintain body temperature at 37°C. The acquisition was performed during 40 minutes with two 15% windows centered on the two peaks 171 keV and 245 keV of ^111^In. All image data were reconstructed and analyzed using InVivoScope (Bioscan, Washington, DC, USA).

### 2.13 *In vivo* antitumor activity of the formulated LPS and MP-LPS in murine models

**Figure 1:**
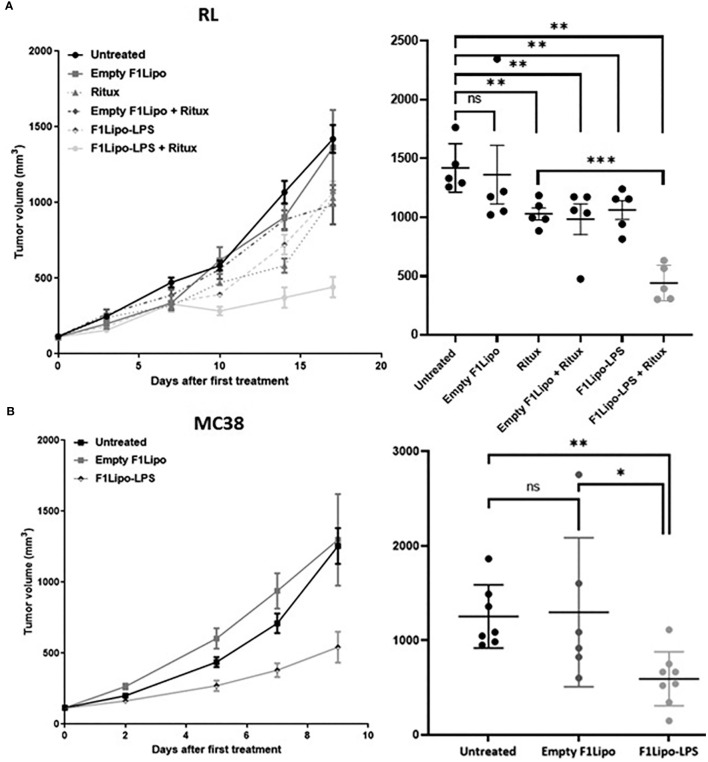
Antitumor activity of F1Lipo-LPS *in vivo*. **(A)** RL tumor cell lines were injected in SCID mice subcutaneously. When tumors reached 100 mm^3^, mice were randomized and treated with Empty F1Lipo or F1Lipo-LPS alone or in combination with rituximab. **(B)** MC38 tumor cells lines were injected in C57BL/6 subcutaneously, when tumors reached 100 mm^3^, mice were randomized and treated with Empty F1Lipo or F1Lipo-LPS. RL and MC38 scatterplot of individual tumor volumes on day 17 and day 9, respectively. Data shown are mean tumor volume values and error bars ± SEM n=5 to 8 mice/group. ns, not significant; *: p<0.05 **: p<0.01, using Mann Whitney t-test for RL and MC38 experiments.

**Figure 2:**
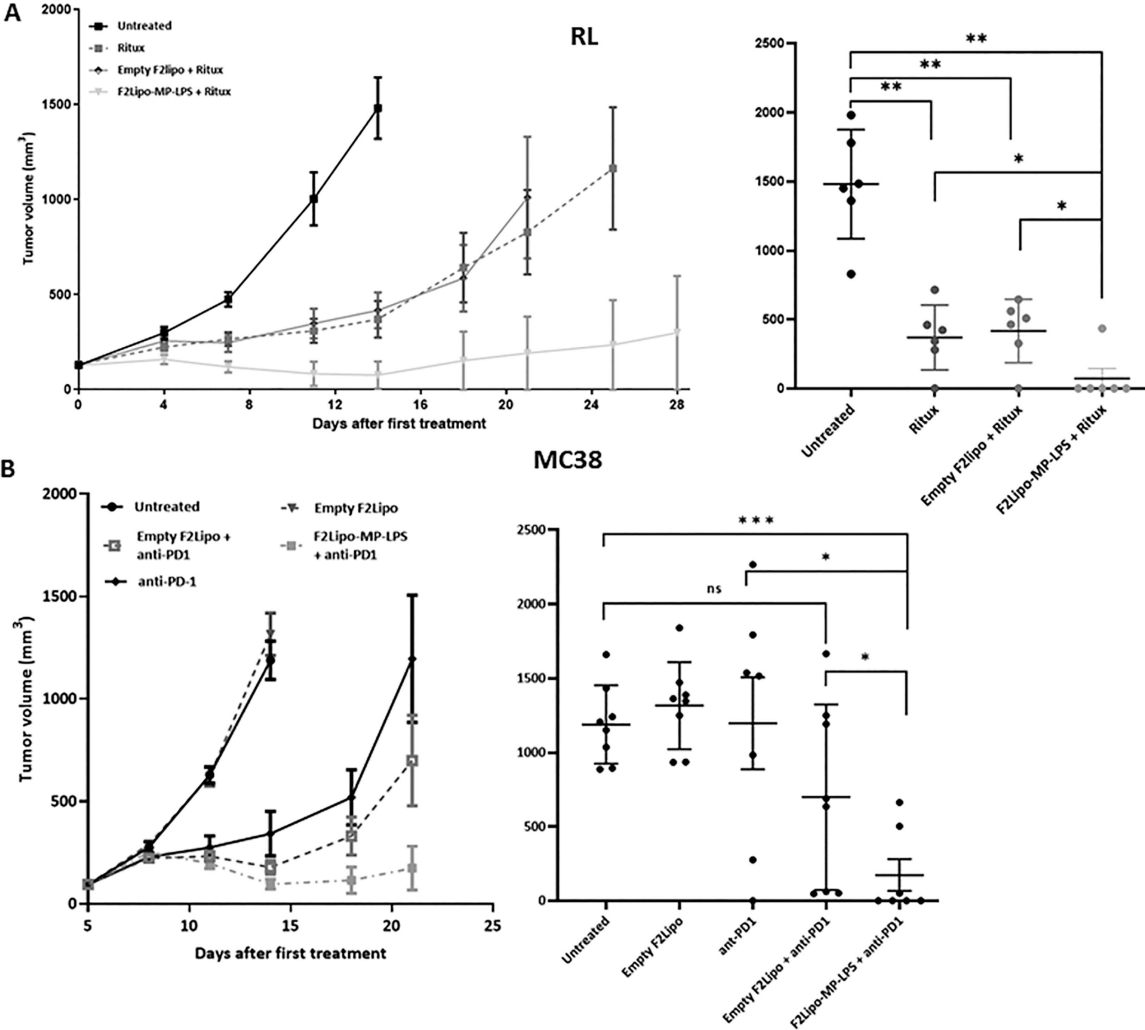
Antitumor activity of F2Lipo-MP-LPS. **(A)** In RL model, F2Lipo-MP-LPS, but not Empty F2Lipo, enhanced the antitumor efficacy of the anti CD20 antibody rituximab. Scatterplot of individual tumor volumes on day 14. **(B)** F2Lipo-MP-LPS but not empty F2Lipo significantly enhanced the antitumor activity of anti-PD1 (p<0.01) in the murine syngeneic colorectal MC38 model. Scatterplot of individual tumor volumes on day 14 for untreated and empty F2lipo mice groups and on day 21 for Ritux and F2lipo-MP-LPS mice groups. Data shown are mean tumor volume values and error bars ± SEM n=5 to 8 mice/group, *: p<0.05 **: p<0.01, ***: p<0.001 using Mann Whitney t-test for RL and MC38 experiments. ns, not significant.

Antitumor activity was analyzed both in immunocompromised and in immunocompetent models. Six-week-old female CB17 severe combined immunodeficient (SCID) mice and C57BL/6 mice purchased from Charles River Laboratories were bred under pathogen-free conditions at the animal facility of our institute. Animals were treated in accordance with the European Union guidelines and French laws for laboratory animal care and use. The animals were kept in conventional housing. Access to food and water was not restricted. This study was approved by the CECCAPP Animal Ethics committee. Continuous health monitoring was carried out on a regular basis, with daily monitoring of clinical symptoms and adverse effects. Animals of an average weight of 20 g were inoculated subcutaneously (SC) with RL, MC38 or A20 of exponentially growing cultures diluted in 0.2 mL of PBS and injected (5.10^6^ cells) into the right flank. When SC tumors reached a median volume of 100 mm^3^ animals were randomized and treatments were initiated. For the K7M2 osteosarcoma model, exponentially growing cultures were diluted in 0.1 mL of PBS and injected (1.10^6^ cells) into the right femur and treatments were initiated 48 hours after implantation. Treatments consisted in a weekly injection of F1Lipo-LPS or F2Lipo-MP-LPS (500 μg/kg) administered intravenously. In the RL model, 30 mg/kg of rituximab was administered weekly intraperitoneally. In the MC38 model, F1Lipo-LPS was administrated as single agent therapy (**Figure 1B**) and F2Lipo-MP-LPS treatment was combined to anti-PD1 treatment (12.5 mg/kg), administered intraperitoneally once per week (**Figure 2B**). Empty liposomes (devoid of LPS) were used as controls. Primary tumor volume (TV) was calculated as TV = 4/3πR^3^. Measurements were performed twice a week.

### 2.14 Impact of F1Lipo-LPS on the immunocompetent C57BL/6 mice immune system

We evaluated the impact of Empty F1Lipo and F1Lipo-LPS on immune subpopulations in immunocompetent C57BL/6 mice bearing the colorectal MC38 model. 24h after the second treatment tumors were collected and dissociated with a mouse tumor dissociation kit (130-096-730, Miltenyi Biotec). To digest tumors, gentle MACS Octo Dissociator (130-096-427, Miltenyi Biotec) was used. After filtration at 100 µm (130-110-917, Miltenyi Biotec) and wash, cell surface markers were stained with the fluorescently labeled antibodies in the dark for 30 min at 4°C. After surface staining, cells were fixed and permeabilized using BD Cytofix/Cytoperm kit (BD, 554714, RRID: AB-2869008) and then labeled with intracellular fluorescently labeled antibodies in the dark for 30 minutes at 4°C. Samples were acquired in Cytek^®^ Aurora flow cytometer with SpectroFlo^®^Software (Cytek^®^ Biosciences). A panel of 30 antibodies was used to evaluate immune infiltration (**Supplementary Table 2**). Gating was performed as shown in Supplementary data (**Supplementary Figures 2A, B**).

### 2.15 Analysis of TLR4-associated pathways

Blood-derived monocytes were provided by the Lyon Blood Bank. Monocytes were pre-differentiated into M1-like cells and M2-like cells by culture for 6 days in RPMI/10% FCS supplemented with either 50 ng/mL GM-CSF (Miltenyi Biotec) and M-CSF (Miltenyi Biotec), respectively. In order to obtain M1 and M2 fully polarized cells, macrophages were cultured for an additional 20 h in presence of IFNγ (50ng/mL, Miltenyi Biotec) and IL-4 (20 ng/mL, Miltenyi Biotec), respectively. Fully differentiated macrophages were exposed for 2 hours to IFN-γ (50 ng/mL), F2Lipo-MP-LPS (100 ng/mL), MP-LPS (100 ng/mL), empty F2Lipo or IL-4 (20 ng/mL).

### 2.16 Rabbit pyrogenicity assay

The rabbit pyrogenicity assay was performed according to the European Pharmacopoeia at the *European Research Biology Center* (ERBC) based in Baugy (France). Female New Zealand white rabbits ranging in weight from 2.0 to 3.3 kg were used throughout this study. Rabbits were given an intravenous dose (1 mL/kg of body weight) of 0.75 ng/kg of LPS, 175 ng/kg of MP-LPS and 1750 ng/kg of F2Lipo-MP-LPS. Rectal temperatures were measured with indwelling rectal thermistors and recorded for 3 h after pyrogen administration. According to the European Pharmacopoeia, the compound is considered pyrogen free if the summed response of the difference between the highest temperature 3 hours post-injection and baseline temperature for three rabbits does not exceed 1.15°C, and fails if the summed response exceeds 2.65°C.

### 2.17 Dog toxicity analysis

Groups of three healthy Beagles received a 30-minutes intravenous infusion of F2Lipo-MP-LPS at increasing doses: 3 μg/kg on day 1, 6 μg/kg on day 8 and 10 μg/kg on day 15. Animals were monitored for general welfare, body temperature and blood samples were drawn prior to administration then 3, 6, 9 and 24 hours after administration. Blood samples were analyzed for liver enzymes *alanine aminotransferase* (ALAT), aspartate aminotransferase (ASAT) and alkaline phosphatase (AP) and blood counts. This study was performed by the Institut Claude Bourgelat (Marcy l’Etoile, France) and approved by the ENVL Animal Ethics Committee.

### 2.18 Statistical analyses

Data were processed using GraphPad Prism 9.0. Mann-Whitney’s test was used to determine statistical differences in *in vivo* experiments, and Two-way ANOVA with Bonferroni *post-hoc* was performed for flow cytometry analysis. Results were expressed post-normalization on 100,000 events taking into account CD45+ viable cells only. P values are shown for relevant statistical differences.

## 3 Results

### 3.1 Liposome characterization

**Figure 3:**
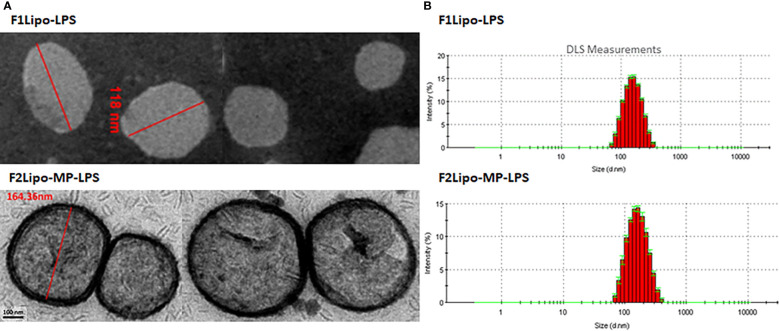
Characterization of liposomal formulation. **(A)** Representative transmission electron microscopy images of F1Lipo-LPS and F2Lipo-MP-LPS liposomal particles after extrusion. **(B)** DLS measurements were performed to determine the size and polydispersity of liposomal particles F1Lipo-LPS and F2Lipo-MP-LPS.

LPS and MP-LPS were formulated in two different liposomal formulations and the size, polydispersity index (PDI), zeta potential of the particles and encapsulation efficiency (EE) were analyzed (**Table 1**). Two major reasons led us to choose the F2 liposomal formulation. First, its composition is identical to that of an amphotericin B lipid complex approved for use to treat fungal infection (Abelcet^®^). Second, the composition of this formulation guarantees a high LPS incorporation as demonstrated by E. Bennett-Guerrero et al. (31). Small unilamellar vesicles (**Figure 3A**) were obtained with a mean size of 145 ± 2.43 nm and 146 ± 4.3 nm for F1Lipo-LPS and F2Lipo-MP-LPS, respectively. For both formulations, a PDI<0.13 indicated a homogeneous population of unilamellar vesicles (**Figure 3B**). As demonstrated by Ruyra et al. (32) and taking into account the amphiphilic structure of LPS, confocal microscopy analysis of fluorescently labeled liposomes containing fluorescently tagged LPS confirmed the presence of encapsulated LPS in the lipid bilayer of the liposome (**Supplementary Figure 3**). **Supplementary Figure 3C** shows the superposition between the fluorescence of Rhodamine B-Liposomes (red) and FITC-LPS (green). As expected from lipid composition, the F2Lipo-MP-LPS formulation was anionic with the zeta potential of -25.07 ± 1.27 mV whereas the pegylated formulation, F1Lipo-LPS, possessed a neutral zeta potential of -2.96 ± 0.3 mV. The F1Lipo-LPS and F2Lipo-MP-LPS present a high EE of 89% and 74%, respectively.

**Table 1:** Composition and characterization of liposomal formulations.

Name	Liposome composition	Size (nm)	PDI	Zeta potential (mV)	EE (%)
**F2Lipo-LPS**	DOPE 54%: DSPE-PEG5000 8%: DSPE-PEG350 8%: Chol 30%	145±2.4	0.09±0.006	-2.96±0.3	89
**F2Lipo-MP-LPS**	DMPC 70%: DMPG 30%	146±4.3	0.12±0.1	-25.7±1.27	74

### 3.2 *In vitro* binding of liposomal formulations to fresh leukocytes

**Figure 4:**
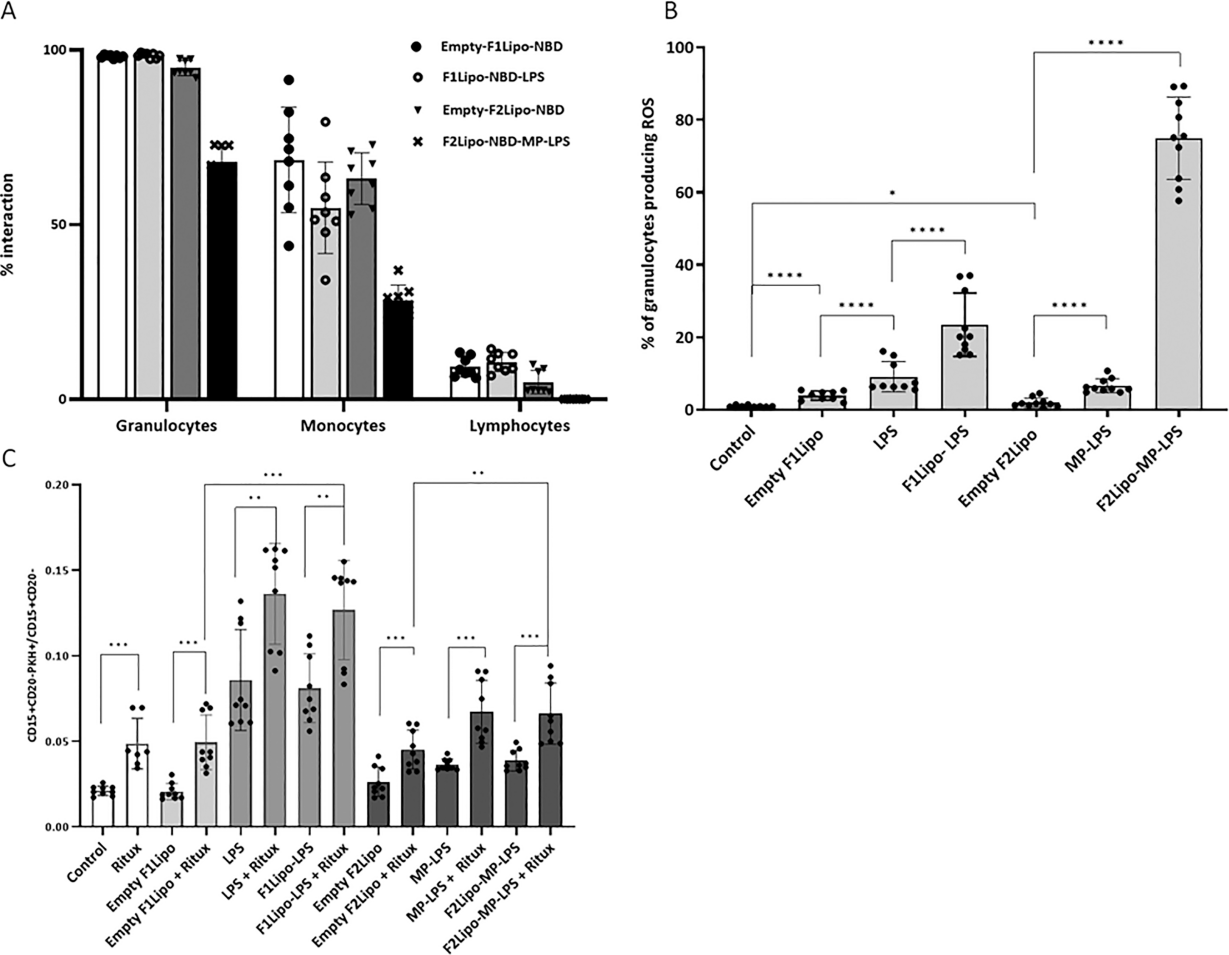
Impact of liposomal formulation on fresh human leukocytes. **(A)** flow cytometry analysis of DNB labeled liposomal formulations binding to fresh human leukocytes. Both liposomal formulations bound to granulocytes and monocytes but very weakly to lymphocytes. **(B)** Effect of Lipo-LPS on the production of reactive oxygen species (ROS) by granulocytes using the Fagoflow method. F1Lipo-LPS and F2Lipo-MP-LPS induced a significant increase of percentage of granulocytes producing ROS in comparison with Empty Liposomes (****p<0.0001, using Mann Whitney t-test/three independent experiments). **(C)** Impact of Lipo-LPS on Antibody-Dependent Cellular Phagocytosis (ADCP). Rituximab-mediated phagocytosis of RL lymphoma cells by fresh human leukocytes was enhanced in the presence of F1Lipo-LPS and F2Lipo-MP-LPS (**p<0.01, using Mann Whitney t-test/three independent experiments). *p<0.05; ***p<0.001.

As shown in **Figure 4A**, the two liposomal formulations bound to both granulocytes and monocytes but very weakly to lymphocytes (less than 10%). More than 95% of granulocytes were stained when incubated with emptyF1Lipo-NBD, F1Lipo-NBD-LPS and emptyF2Lipo-NBD. F2Lipo-MP-LPS binding to granulocytes (68.05%) and monocytes (28.20%), although less, remained substantial. The flow cytometry analysis gating procedure is shown in **Supplementary Figure 4**. A similar observation was made with murine splenocytes, with F1Lipo-LPS-FITC binding to monocytes (85.7% staining), macrophages (52.3% staining) and neutrophils (56.9% staining) but not to CD4+ T cells, CD8+ T cells and CD19+ B cells (**Supplementary Figure 5**).

### 3.3 Induction of phagocytosis and enhancement of ADCP

Analysis of the reactive oxygen species (ROS) content using the FagoFlow method showed that formulated LPS and MP-LPS significantly enhanced the production of ROS by granulocytes, a key step in the phagocytic process, in comparison to unformulated LPS and MP-LPS, respectively. This increase is much greater when granulocytes were incubated with F2Lipo-MP-LPS (**Figure 4B**). The difference in composition and therefore in zeta potential between the two liposomal formulations may be the cause of this observation. It should be noted that the sizes of the F1lipo and F2Lipo liposomes are equivalent. The different molecules were also evaluated in an *in vitro* ADCP assay in which fresh human phagocytes were exposed to CD20 positive non-Hodgkin RL cells in the presence of the anti-CD20 antibody rituximab. As shown in **Figure 4C**, F1Lipo-LPS and F2Lipo-MP-LPS were found to possess ADCP-inducing activity *per se* and to significantly enhance the ADCP activity of rituximab *in vitro*.

### 3.4 F1Lipo-LPS preferentially localizes in spleen

**Figure 5:**
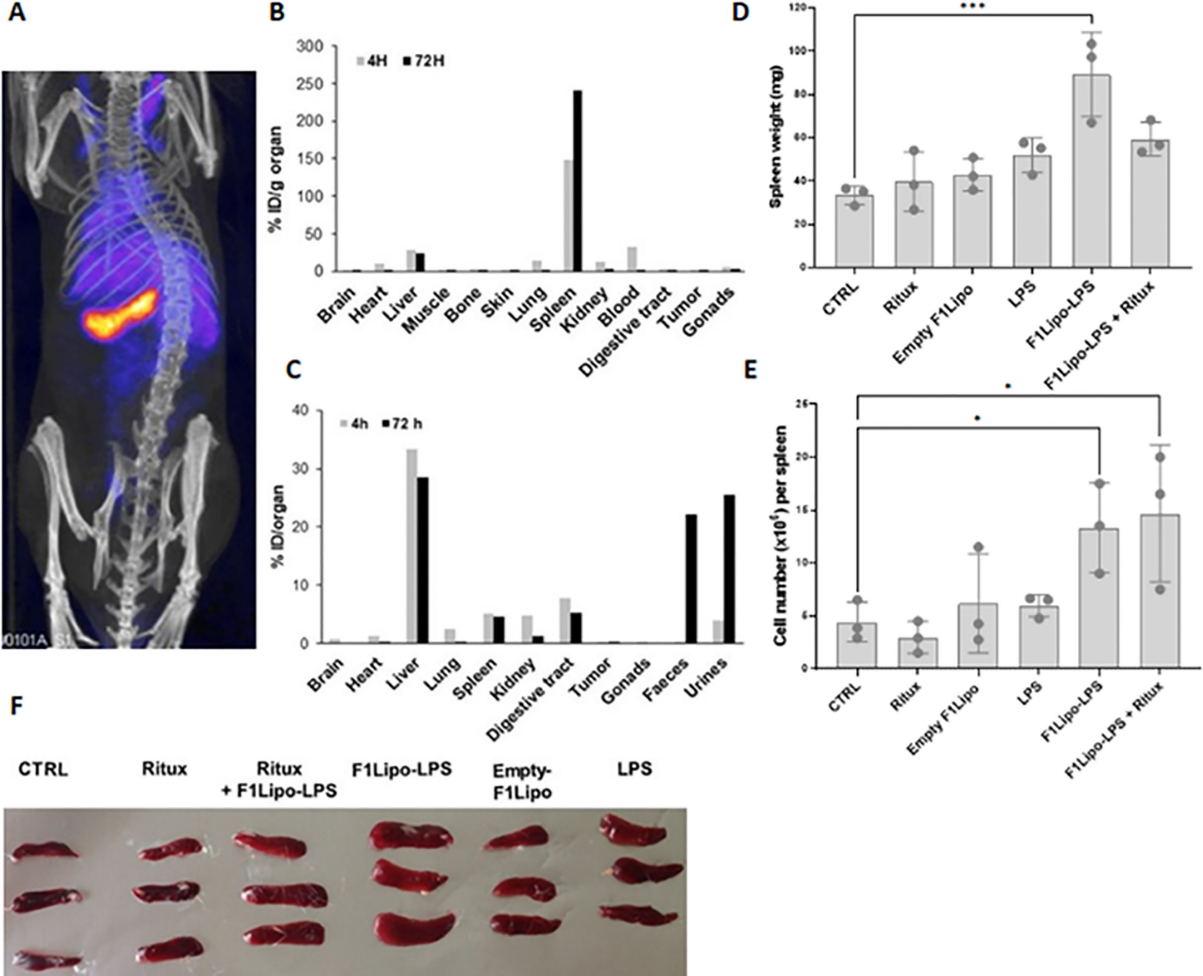
F1Lipo-LPS preferentially localizes in spleen. **(A)** SPECT-CT scan imaging of C57BL/6 mice bearing established colorectal MC38 tumors intravenously injected with ^111^In-labeled F1Lipo-LPS; **(B–C)** Distribution of ^111^In in the tissues. On a per gram basis, binding to spleen exceeded hepatic binding but the total amount bound to liver was greater than that bound to spleen. ^111^In found in MC38 tumors was low. **(D)** Impact of F1Lipo-LPS on spleen weight, **(E)** cellularity and **(F)** size in RL-bearing SCID mice (3 mice/group) injected once a week for 3 weeks with rituximab (Ritux; 30 mg/kg), unformulated LPS (LPS, 500 μg/kg), F1Lipo-LPS (500 μg/kg) or Empty Lipo. Mice not treated were used as control. Spleens were harvested 2 days after the last treatment, dissociated into single-cell suspensions and leukocytes counted by Trypan blue exclusion. Results are the mean ± SD of 3 mice per group. *p<0.05, ***p<0.001.

Biodistribution analyses in mice bearing established colorectal MC38 tumors injected with ^111^In-labeled F1Lipo-LPS demonstrated both early and prolonged localization in spleen (**Figure 5A**). On a per gram basis, binding to spleen exceeded hepatic binding (**Figure 5B**) but the total amount bound to liver was greater than that bound to spleen (**Figure 5C**). Of note the total amount found in MC38 tumors was low, both on a per gram basis and as a percentage of the total amount administered. As shown in **Supplementary Figure 6**, ^111^In-labeled F2Lipo-MP-LPS exhibits a similar binding to spleen and liver on a per gram basis. This can be explained by the negative charge of the liposomal particles of the F2Lipo-MP-LPS formulation (zeta potential of -25.07 ± 1.27 mV). In the RL model, repeated exposure of SCID mice to F1Lipo-LPS was associated with an increase in spleen weight, cellularity and volume (**Figures 5D–F**). Overall, these data support a strong splenic uptake of F1Lipo-LPS with a systemic activation of the immune system.

### 3.5 Impact of F1Lipo-LPS on the immunocompetent C57BL/6 mice immune system

**Figure 6:**
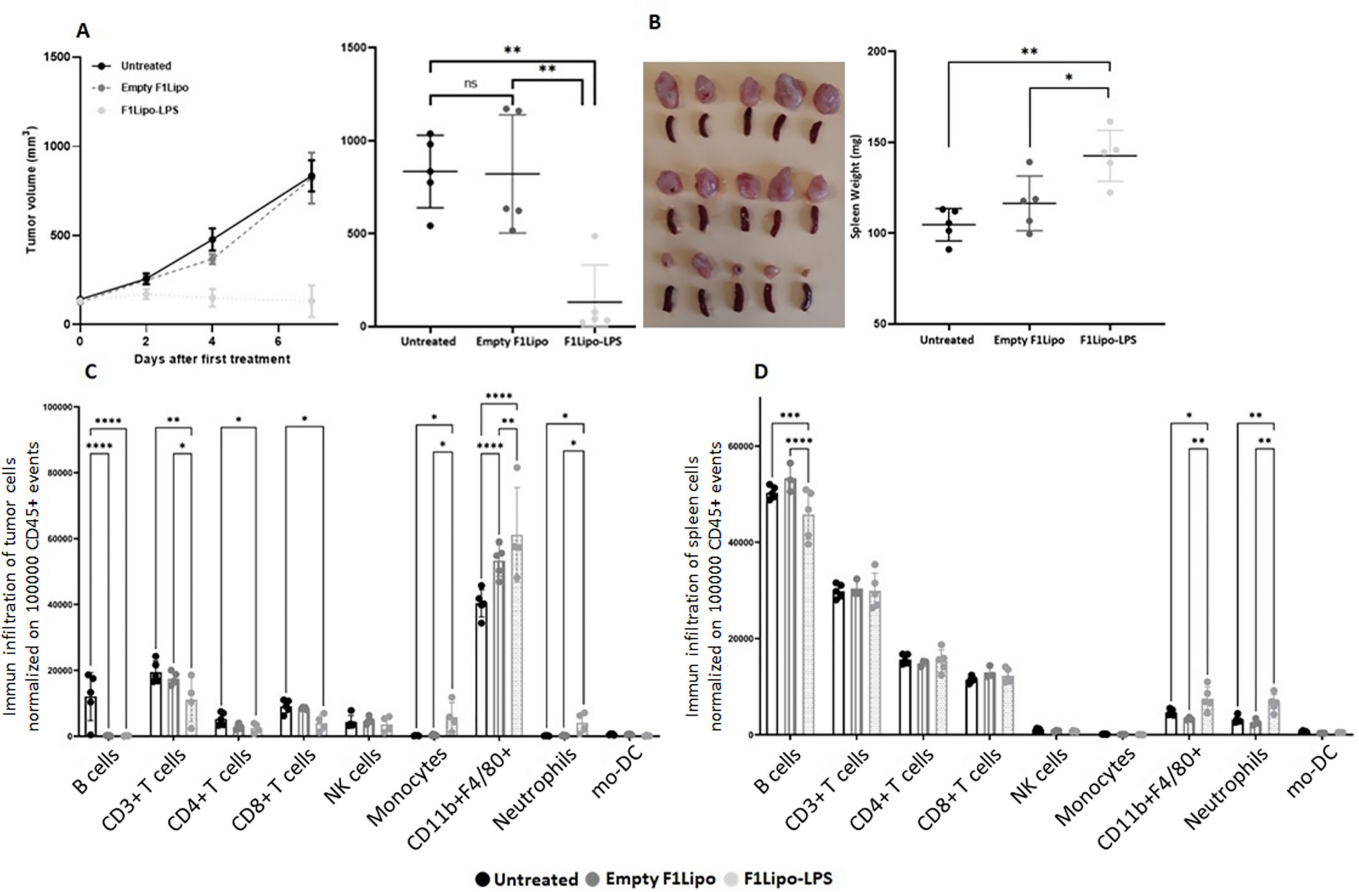
Impact of F1Lipo-LPS on the murine immune system by Cytek^®^ Aurora. MC38 tumor cell lines were injected in C57BL/6 mice subcutaneously. When tumors reached 100 mm^3^, mice were randomized and treated once a week for 2 weeks with Empty F1Lipo or F1Lipo-LPS. **(A)** Tumor growth curves over time in each group. Data shown are mean tumor volume (mm^3^) ± SEM (n=5 mice/group); **: p>0.01, using Mann Whitney t-test. **(B)** 24h after the second weekly treatment administration, tumors and spleen were collected, tumor volumes were measured, and spleens were weighed. Data shown are mean tumor volume and spleen weight values, error bars are ± SD n=5 mice/group, *: p<0.05, **: p>0.01, using Mann Whitney t-test. **(C, D)** Immunophenotypic analyses in tumors and spleens. Flow cytometry analysis was performed 24h after the second weekly treatment administration. Histograms for each immune population were established after normalization of the number of cells of interest in 100,000 CD45+ viable events. Significant decreases and increases were assessed by a two-way ANOVA statistical test, with Bonferroni *post-hoc* test. n = 4 to 5 tumors per group, and n=3 to 5 spleens per group, for each *: p<0.05, **: p<0.01, ***: p<0.001, ****: p<0.0001.

Cytometry analyses (Cytek^®^ Aurora) were performed to characterize peripheral and tumor-infiltrating immune cell populations in C57BL/6 mice bearing established colorectal MC38 tumors exposed to vehicle, Empty F1Lipo or F1Lipo-LPS once weekly. Using a panel of 30 markers, 20 independent cell clusters were identified (**Supplementary Figures 7A, B**). Tumors and spleens were harvested 24 hours after the second administration, when the impact on tumor growth was already significant (**Figures 6A, B**). At tumor site, significant increases in neutrophils, monocytes and macrophages (p<0.05) were observed in the Lipo LPS-exposed group compared to controls, associated with a significant decrease in B cells as well as CD4+ and CD8+ T cells (p<0.05) (**Figure 6C**). Analysis of TLR4 expression identified in tumors of mice exposed to F1Lipo-LPS, but not to controls, a subpopulation of CD11b+F4/80 macrophages highly expressing TLR4 and co-expressing CD19 (**Supplementary Figure 7A**). In the spleen, significant differences were an increase in macrophages and neutrophils (p<0.05 and p<0.01, respectively) (**Figure 6D**). Moreover, MHCII+SIRPα-F4/80+CD11b+ macrophages increased after F1Lipo-LPS exposure (**Supplementary Figure 7B**). This subpopulation is associated with a well-established role in tumor regression (33). Subpopulation analyses (**Supplementary Figure 1**) also showed an increase of naïve CD8+ cells in spleen and a decrease of these cells in tumors (**Supplementary Figures 7A, B**). Monocyte-derived dendritic cells were found to be decreased after exposure to Lipo LPS both in tumor and in spleen (**Figures 6C, D**).

### 3.6 Antitumor activity of F1Lipo-LPS in murine models

In immunodeficient mice bearing RL tumors F1Lipo-LPS and the anti CD20 antibody rituximab were administered by weekly intravenous and intraperitoneal injections, respectively. Single agent F1Lipo-LPS had no or modest antitumor activity *per se* but significantly enhanced the antitumor activity of the tumor targeting antibody. As shown in **Figure 1A**, F1Lipo-LPS, but not Empty F1Lipo, enhanced the antitumor efficacy of the anti CD20 antibody rituximab in the RL human NHL model (p<0.01). A similar observation was made in the non-Hodgkin’s lymphoma model Granta exposed to GA101, another CD20-targeted antibody (**Supplementary Figure 8**). The F1Lipo-LPS also displayed a significant intrinsic antitumor activity in immunocompetent mice. In the murine syngeneic colorectal MC38 model, F1Lipo-LPS induced as monotherapy significant tumor growth delay (p<0.01) (**Figure 1B**). Similar observations were made in the orthotopic osteosarcoma model K7M2 (p<0.05) (**Supplementary Figure 9A**) and in the syngeneic NHL model A20 (p=0.0571) (**Supplementary Figure 9B**). Additionally, F1Lipo-LPS was found to enhance the efficacy of anti-PD-1 antibody in the MC38 model (**Supplementary Figure 10**).

### 3.7 MP-LPS production and quality control

The *sine qua non* condition before considering phase I clinical trials in man is to substitute the highly toxic LPS with a detoxified LPS. Backed by our expertise and experience we produced MP-LPS through chemical modification of a purified LPS extract using alkali and acid treatments. Structural quality control analyses by MALDI-MS on the final product confirmed the efficacy of the detoxification process on the lipid A moiety without modification of the oligosaccharide domain (**Table 2**). Further quality control analyses demonstrated the high level of solubility of MP-LPS in water (>10mg/ml) leading to the formation of small supramolecular aggregates of about 30nm. LC-MS and spectrophotometry (absorbance at 260 nm) analysis also demonstrated the high level of purity of the MP-LPS containing less than 0.5% proteins and 0.2% of nucleic acids (**Table 2**). Finally, *in vitro* assays on HEK-Blue TLR4/TLR2 cells showed its capacity to strongly activates the TLR4 (EC50 = 20ng/ml) but not the TLR2 pathway (EC50>10µg/ml, extrapolated value of about 200µg/mL).

**Table 2:** Quality control of MP-LPS.

Parameters	Values
**Molecular weight**	About 3500 Da
**Solubility**	>10 mg/mL ( at 10000 g)
**Nucleic Acids content**	Undetectable at 20μg/μL, LOD 0.2%
**Protien content**	< 0.5% w/w
**HEK-Blue EC50 hTLR2/EC50 hTLR4**	200μg/mL/20 ng/mL=1.10^4^
**Aggregates Size (in water, by DLS)**	20-30 nm

### 3.8 Validation of MP-LPS

**Figure 7:**
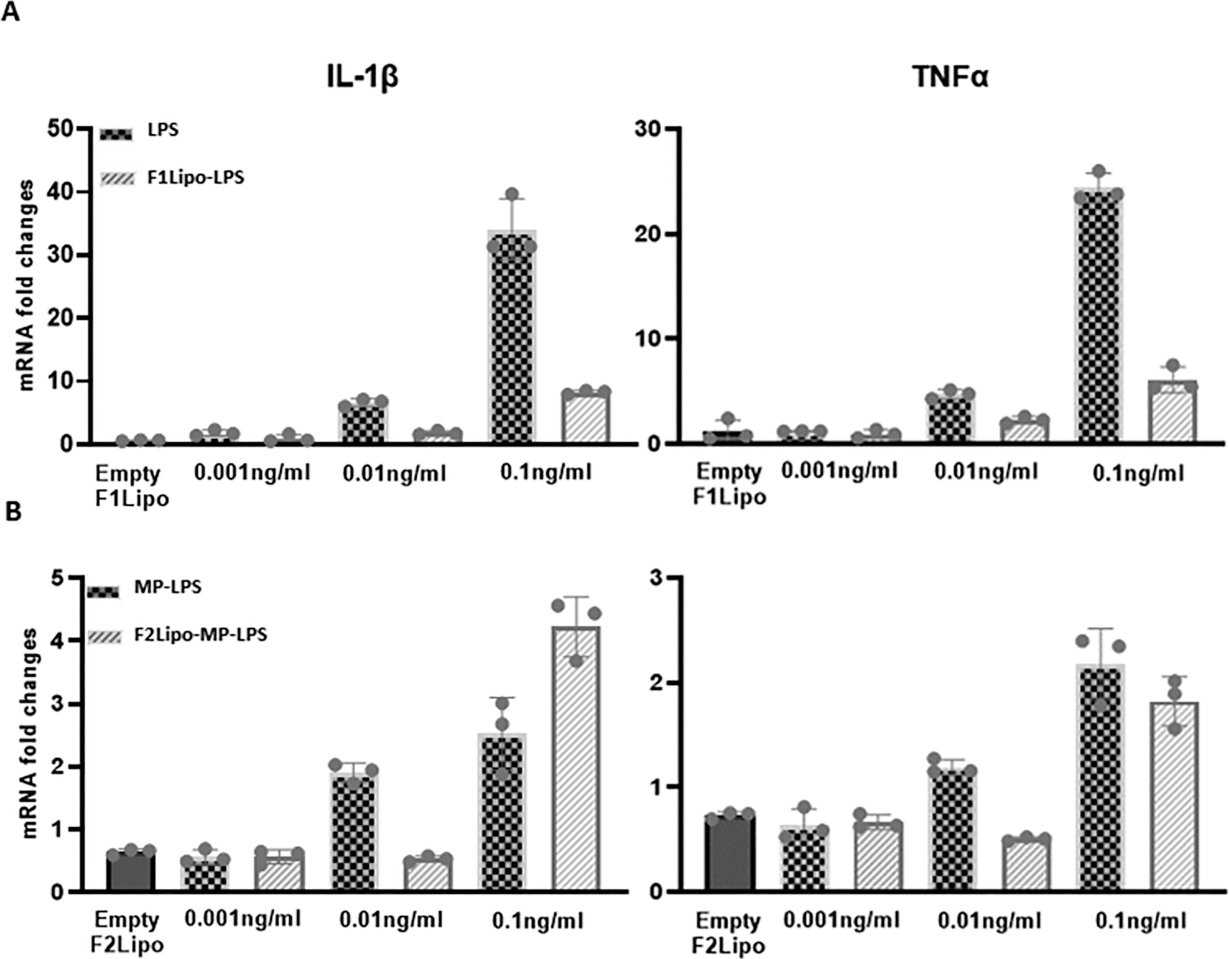
Monocyte activation tests comparing unformulated LPS and Lipo-LPS on fresh human monocytes. **(A)** Fresh human peripheral blood monocytes were exposed to various concentration of F1Lipo-LPS or unformulated LPS for 90 minutes and assessed for IL-1β and TNFα expression by RT-PCR. **(B)** The analysis described in **(A)** is carried out with unformulated MP-LPS or F2Lipo-MP-LPS.

To validate the impact of both the detoxification process and the liposomal formulation on the toxicity profile of MP-LPS, gene expression of pro-inflammatory cytokines was analyzed in fresh human monocytes exposed to increased concentrations (up to 0.1 ng/ml) of LPS or MP-LPS, formulated or not in liposomes (**Figure 7**). The mRNA content for pro-inflammatory cytokines IL-1β and TNFα were found to be significantly higher in monocytes stimulated with the unformulated LPS (**Figure 7A**) compared to equivalent doses of unformulated detoxified LPS (**Figure 7B**). This confirms that the chemical detoxification process significantly reduces the inflammatory activity of LPS. Interestingly, the liposomal formulations were also found to significantly reduce the inflammatory activity of both the LPS and MP-LPS although this activity was found to remain higher with F1Lipo-LPS compared to F2Lipo-MP-LPS. Rabbit pyrogen tests confirmed these observations (**Supplementary Figure 11**). MP-LPS presented a pyrogenic activity significantly lower than LPS as characterized by a 233-fold higher Maximum non-pyrogenic dose (MNPD) (175ng/kg vs 0.75ng/kg). MP-LPS was also found to present a pyrogenic activity 2.5-fold lower than the MPL (175ng/kg vs 73ng/kg – Data not shown), a non-GMP equivalent of the TLR4 agonist molecule from GSK (MPL) currently used in 4 human vaccines. Interestingly, the liposomal formulation was found to further reduce the pyrogenic activity of MP-LPS 10-fold (MNPD = 1750 ng/kg) while it only reduced the pyrogenic activity of LPS 2-fold (MNPD = 1.5 ng/kg). These results show that the liposomal encapsulation and chemical detoxification process can synergize to reduce the toxicity of LPS and suggest that the F2Lipo-MP-LPS is significantly less toxic than the MPL from GSK.

### 3.9 F2Lipo-MP-LPS toxicity study in dog

A single dose escalation toxicity study of systemically administered Liposomal chemically detoxified LPS has been conducted in a group of 3 dogs to evaluate its safety and innocuity. As shown in **Table 3** systemically administered F2lipo-MP-LPS was well tolerated in dogs. The main clinical effects detected were transient hyperthermia and tremors (resolved 6 hours after administration) observed in 1 dog at the 3 and 10 µg/kg dose levels, and in 2 dogs at the 6 µg/kg dose level. Biological analyses showed transitory elevation of phosphatase alkaline levels in one of the three animals at each dose level but not ALAT nor ASAT serum transaminases. All animals presented transitory leukopenia 3 hours after administration with normalization at 24 hours post-administration.

**Table 3:** Groups of 3 healthy beagles received a 30-minute intravenous infusion of F2Lipo-MP-LPS.

Dose (microg/kg)	Infusion time (min)	Transitory hyperthermia/tremors	Increased ALAT Or ASAT levels	Increased PA levels	Transitory Leukopenia
**3**	30	1/3	0/3	1/3	3/3
**6**	30	2/3	0/3	1/3	3/3
**10**	30	1/3	0/3	1/3	3/3

Dose levels were increased weekly, with a starting dose of 3 μg/kg on day 1, 6 μg/kg on day 8 and 10 μg/kg on day 15. Bloods were drawn then 3, 6, 9 and 24 hours after infusion.

### 3.10 Impact of F2Lipo-MP-LPS on polarized macrophages

**Figure 8:**
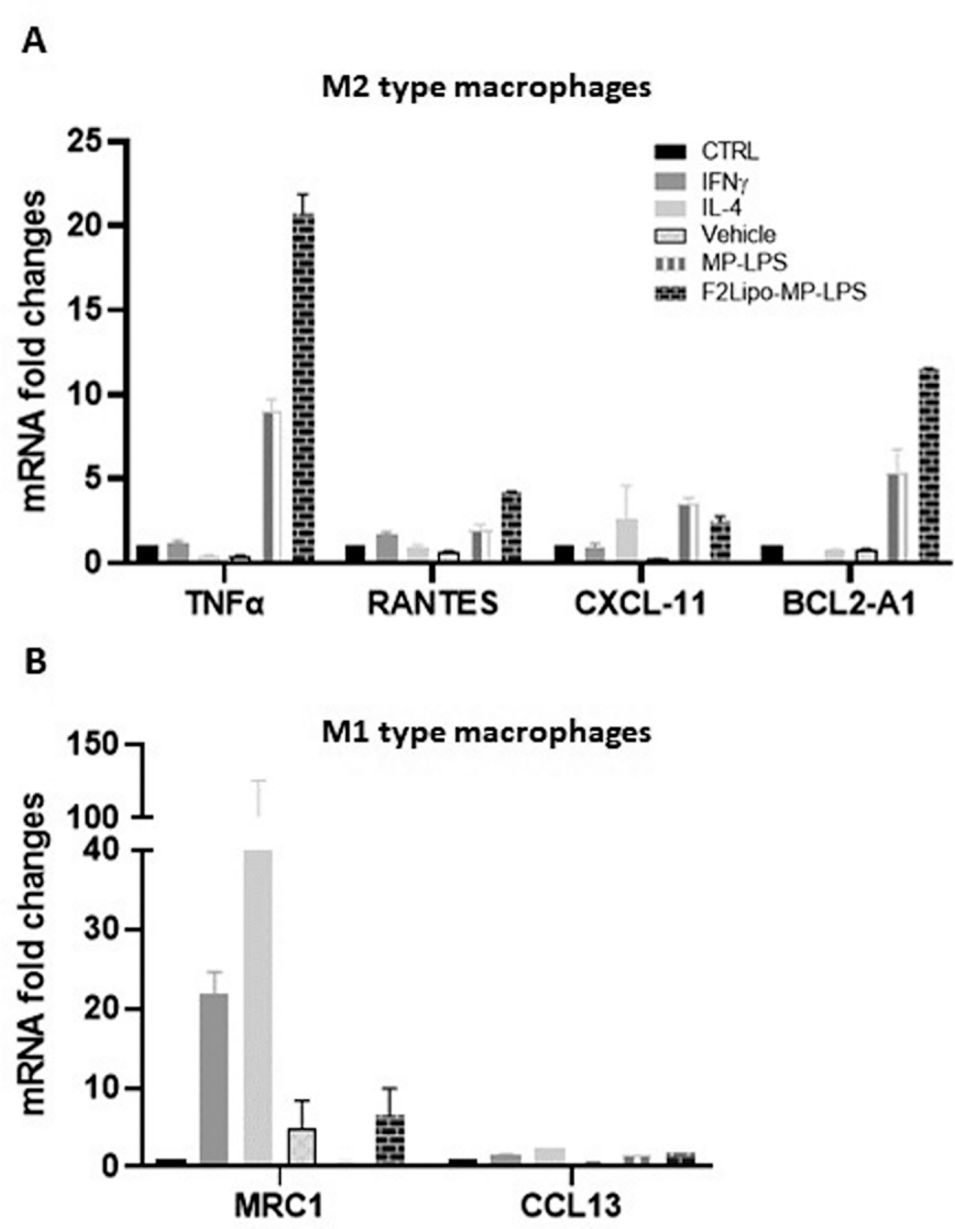
F2Lipo-MP-LPS induces M2-M1 phenotype switch. **(A)** qPCR expression analysis of M1 associated genes (TNF-α, RANTES, CXCL11 and BCL2-A1) in fully differentiated M2 human macrophages and **(B)** M2 associated genes (MRC-1 and CCL13) in fully differentiated M1 human macrophages following 2 hours of *in vitro* stimulation with F2Lipo-MP-LPS or MP-LPS (100 ng/ml), IFN-γ (50 ng/ml), IL-4 (20 ng/ml), empty F2Lipo, or only medium as controls.

The M1 macrophage phenotype is characterized by the expression of high levels of pro-inflammatory cytokines, production of reactive nitrogen and oxygen intermediates, promotion of Th1-based responses and tumoricidal activity (34). In contrast, M2 macrophages are characterized by their involvement in tissue modelling, angiogenesis, and immune suppression, thereby accelerating tumor promotion (35). M2 reprogramming towards a M1 phenotype is a promising approach for improving the efficacy of anti-cancer therapies (36, 37). We evaluated the potential of F2Lipo-MP-LPS to impact the polarization of M1 and M2 macrophages. We analyzed the expression of M1 and M2 associated genes in M1 and M2-polarized human macrophages exposed to the unformulated or formulated chemically detoxified LPS, or to IFNγ,IL-4 or empty liposomes as controls. Results revealed that M1 pro-inflammatory gene expression was remarkably increased in M2 macrophages stimulated with the free MP-LPS but especially with the F2Lipo-MP-LPS, as evidenced by the transcriptional induction of TNF-α, RANTES, CXCL11 and BCL2-A1 (**Figure 8A**). This demonstrates that the chemically detoxified LPS can induce a polarization reversion of human M2 macrophages toward M1 phenotype, and that the liposomal formulation enhances this effect. Both Free MP-LPS and F2Lipo-MP-LPS were also found to reduce the expression of M2-associated genes (MRC-1 and CCL13) in M1 macrophages (**Figure 8B**).

### 3.11 Impact of F2Lipo-MP-LPS on the expression of genes involved in TLR4 pathways

**Figure 9:**
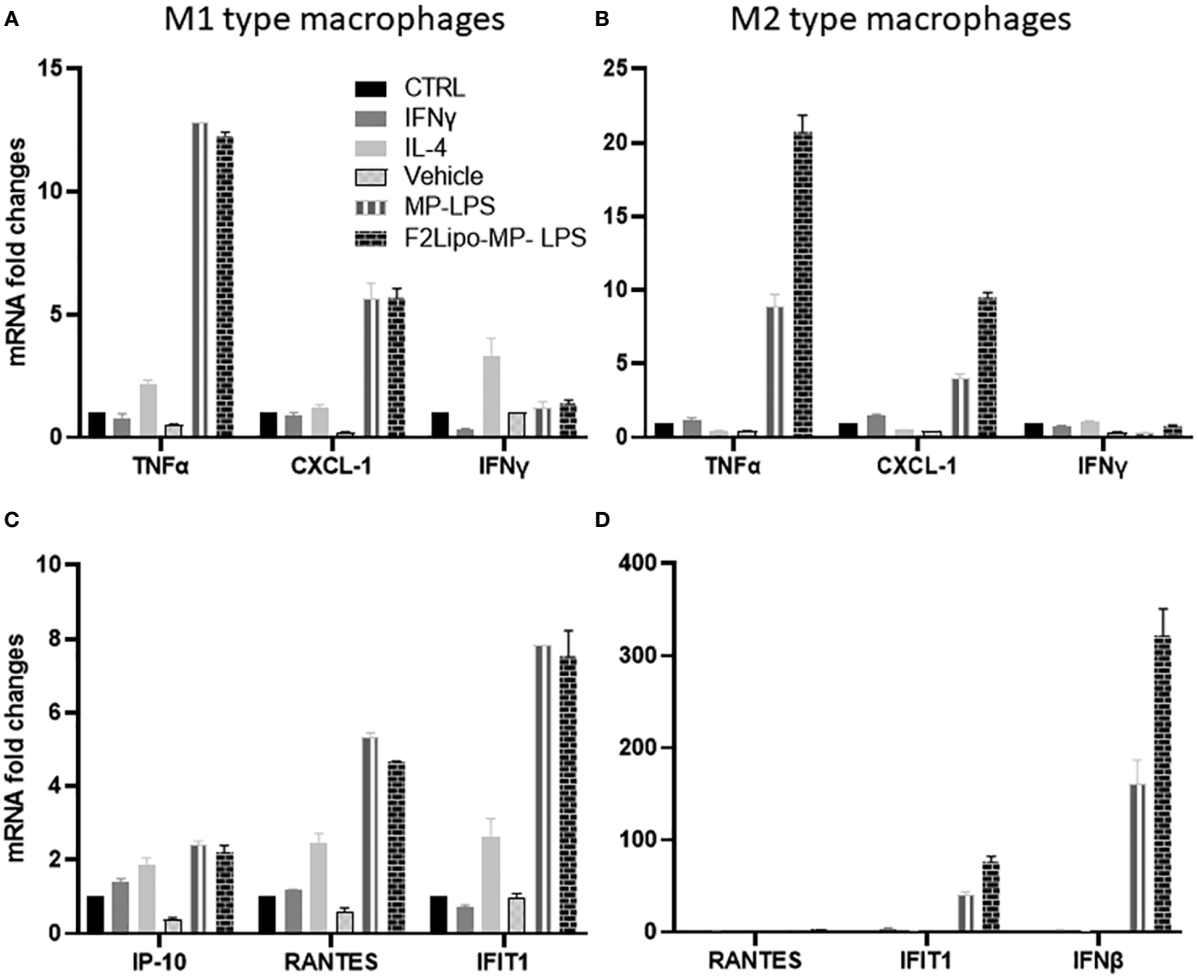
Activation of genes involved in MyD88- and TRIF-dependent pathways. Fully differentiated M1 and M2 human macrophages were exposed for 2 hours to F2Lipo-MP-LPS or MP-LPS (100 ng/ml), IFN-γ (50 ng/ml), IL-4 (20 ng/ml), empty F2Lipo, or medium as controls. **(A, B)** qPCR expression analysis of genes involved in MyD88-dependent pathway in in fully differentiated human M1 and M2 type macrophages. **(C, D)** qPCR expression analysis of genes involved in the TRIF-dependent pathway in fully differentiated M1 and M2 type macrophages.

We explored the activation of the classical (MyD88) and non-canonical (TRIF) TLR4 signaling pathways in human M1 and M2 macrophages exposed to the unformulated or formulated MP-LPS. Both products were found to moderately activate the classical TLR4 pathway in M1 and M2 macrophages, characterized by slight increase of the expression of Myd88-dependent transcripts TNFα, CXCL-1 and IFNγ compared to control groups (**Figures 9A, B**). However, both the unformulated or formulated MP-LPS were found to strongly activate the non-canonical TLR4 signaling pathway, characterized by very high expression of TRIF dependent factors (Rantes, IP-10 and IFIT1) in M1 but especially in M2 macrophages (**Figures 9C, D**). Interestingly, the expression of the TRIF dependent factors was higher in M2 macrophages stimulated with the formulated MP-LPS than with the free MP-LPS. These results show that the MP-LPS preferentially activates the non-canonical TLR4 pathway in M1 and M2 macrophages, which is further increased by the liposomal formulation.

### 3.12 Antitumor activity of F2Lipo-MP-LPS in murine models

As previously described F2Lipo-MP-LPS was administered by weekly intravenous injections to tumor-bearing mice. In immunodeficient mice bearing RL tumors and in the same way as F1Lipo-LPS, the chemically detoxified formulation (F2Lipo-MP-LPS) significantly enhanced the antitumor activity of rituximab (p<0.01), with 5 out of 6 mice showing complete remission vs 1 out of 6 in the group of mice only treated with rituximab +/- empty liposomes (**Figure 2A**). F2Lipo-MP-LPS significantly enhanced the antitumor activity of anti-PD1 (p<0.01) in the murine syngeneic colorectal MC38 model with 6 of 8 mice showing complete remission (**Figure 2B**).

## 4 Discussion

In the present study, we sought to develop a systemically active and tolerated LPS-Based TLR4 agonist by investigating the impact of liposomal formulations and of an innovative detoxification process on the immunostimulatory, antitumor activities and tolerance profile of LPS. We first examined the impact of a liposomal formulation on LPS purchased from Sigma-Aldrich (F1Lipo-LPS) using a liposomal content developed in our laboratory. Liposomal formulations offer several potential advantages for the treatment of cancer. As opposed to conventional agents, liposomal formulations have prolonged half-life, avoiding acute peak/through effects as well as closely repeated injections of the active compound (38, 39). Caron et al. have shown that the functional properties of circulating monocytes and dendritic cells in blood was correlated with systemic clearance in the case of PEGylated liposomal doxorubicin (40). Additionally, liposomal formulations have a different tissue distribution than that observed with conventional formulations. “Stealth liposomes” containing PEG have been shown to have reduced interaction with the immune system (41). Additionally, some lipids have been identified to activate the innate immune system in the context of peptide vaccine delivery (42). Some authors have suggested that liposomes may be interesting vectors for the safe administration of immunotherapeutic agents (43). We demonstrated that LPS can be easily formulated in liposomes due to their amphiphilic nature, leading to the production of small unilamellar vesicles with small size (144nm), a low polydispersity and high encapsulation rates (89%). The formulation of LPS in PEGylated liposomes was found to not impair their capacity to bind to human and murine peripheral blood monocytes and neutrophils nor modify their biodistribution. We indeed observed a preferential localization of F1Lipo-LPS in phagocyte-rich tissues such as the liver and the spleen, as was previously described with unformulated LPS (44). This was associated with an increase in spleen weight, cellularity supporting a strong splenic uptake of F1Lipo-LPS with a local activation of the immune system. We assume that the presence of LPS in the lipid bilayer of the liposomes, as demonstrated by our confocal microscopy analysis, but not inside the liposomes, may explain why the liposomal formulation did not affect the biodistribution of LPS.

Our results also showed that F1Lipo-LPS possesses potent anti-tumor activities in immunocompetent models and strong adjuvant effects in combination with therapeutic monoclonal antibodies in immunodeficient models. This is in keeping with the impact observed on the immune system, namely the activation of monocytes and neutrophils *in vitro*, as well as the recruitment at tumor site of several innate cells, notably TLR4+ macrophages and neutrophils observed in mice exposed to F1Lipo-LPS. This is in line with previous studies which showed that macrophages require TLR4 expression to migrate from the circulation to the tumor microenvironment (45). Of note, the increased macrophage infiltration observed in tumors corresponds to CD11b+F4/80+ cells which also co-expressed CD19, while the B cell content was strongly decreased. This observation is in keeping with previous observations showing generation of macrophage populations from pre/proB cells, with antitumor properties (46, 47). Thus, we may hypothesize that Lipo-LPS facilitates the generation of macrophages with strong anti-tumor potential. Moreover, we showed that F1Lipo-LPS can significantly enhance the ADCP activity of rituximab which is in keeping with its strong adjuvant effects in combination with rituximab observed in immunodeficient mice. These data, as well as the impact of F1Lipo-LPS on spleen cellularity and size, suggest that Lipo LPS behaves as a potent systemic activator of the innate immune system.

Analysis of cell populations in immunocompetent models, however, showed a decreased content in total T cells, CD4+ and CD8+ cells in tumor site, as well as monocyte-derived dendritic in both in tumor and in spleen. The reduced T cell infiltration was unexpected as LPS and Liposomal LPS were previously found to lead to an efficient activation and recruitment of the adaptive immune responses (48, 49). However, it must be emphasized that our analysis was performed at a stage where the tumor volume was already significantly reduced in mice receiving Lipo LPS in comparison to controls. It is therefore possible that cytotoxic effector cells have been recruited earlier during the process but are no longer present at the time of this analysis. As LPS is classically considered to induce dendritic cell activation (50), our observation that monocyte-derived dendritic cells are reduced in responding tumors may also be attributed to the fact that the tumor control had already reached a stage at which recruitment of DC cells is no longer required. Indeed, there are very little data in the literature in which the immune infiltrate was analyzed longitudinally after an acute event or a therapeutic intervention. In the context of myocardial infarction, Rusinkevich et al. showed that the local immune infiltrate evolved rapidly, with a peak total leukocytic infiltration on day 3 followed by a reduced T cell content (51). Additional experiments specifically investigating the dynamics of the tumor immune infiltrate would be required to validate this hypothesis.

A key issue in the systemic activation of the innate immune system with a TLR4 agonist is the possibility to obtain an antitumor response with manageable toxicity. In agreement with previous studies (48), the encapsulation of LPS in liposomes was found to decrease its inflammatory activity as observed in MAT assays, but only reduces its pyrogenic activity by a factor 2. In contrast, an innovative chemical detoxification process was found to reduce the pyrogenic activity of the native molecule by a factor >200 associated with a more significant reduction of its pro-inflammatory activity compared to liposomal formulation.

Taking advantage of the innovative detoxification process, we sought to evaluate in a second part of our study, the impact of a liposomal formulation on a detoxified LPS (F2Lipo MP-LPS) using a clinically approved liposomal content. We first demonstrated that like unmodified LPS, detoxified LPS can also be easily incorporated in the lipid bilayer of liposomes due to the conservation of the oligosaccharide part of the native molecule preserving its amphiphilic nature. We showed that the liposomal formulation further improved the tolerance profile of the detoxified LPS characterized by a significant decrease of its inflammatory activity and reduction of its pyrogenic activity by a factor 10. In addition, while LPS were described to induce severe sepsis symptoms at 0.1 to 2µg/kg doses (52, 53), F2Lipo-MP-LPS was found to be well tolerated in dogs in intravenous administration up to 10µg/kg.

We assumed that the better tolerance profile of the F2Lipo-MP-LPS may be related to its capacity to skew the activation of TLR4 toward the TRIF pathway as previously observed with other LPS derivatives (54). Similarly, Watanabe et al. showed that liposomal formulation can reduce the inflammatory activity of LPS by preferentially inducing the activation of the TRIF-dependent signaling pathway independently of CD14 (48). We have indeed observed that detoxified LPS preferentially activates the non-canonical TLR4/TRIF pathway in human macrophages, and that the liposomal formulation enhances this effect. This is in line with the data of Watanabe et al. who showed that liposomal formulation can reduce the inflammatory activity of LPS by preferentially inducing the activation of the TRIF-dependent signaling pathway, independently of CD14 (48). This enables the production of type-1 interferons, expected to facilitate the induction of adaptive immune responses, while reducing the production of pro-inflammatory cytokines which are responsible for the pyrogenic and toxic effects of LPS. It however remains to be determined whether this TLR4 activation profile observed with the F2Lipo-MP-LPS is secondary to the internalization of LPS which binds to intracellular TLR4 or whether the liposomal formulation impacts on the binding to cell surface TLR4. Internalization of LPS has been shown to involve LPS binding protein (LBP) (55). Both TLR4 and LPS have been shown to traffic through the Golgi apparatus, suggesting that internalized LPS may generate intracellular signalization (56).

We finally demonstrated that the better tolerance profile of F2Lipo-MP-LPS was not associated with a decrease of its efficacy. Our data indeed showed that the detoxified LPS formulated in liposomes conserves a potent antitumor activity *per se* and seems even more efficient than F1Lipo-LPS to enhance the antitumor activity of rituximab in the human RL lymphoma model and of the anti-PD1 in the syngeneic colorectal MC38 model leading to higher rates of complete tumor regressions. Finally, we demonstrated that the detoxified LPS can induce a polarization of human M2 macrophages towards an M1 phenotype (57, 58), an effect that is further enhanced by the liposomal formulation. The M2-M1 phenotype switches, induced by F2Lipo-MP-LPS was confirmed by the remarkably elevated level of M1 pro-inflammatory genes expression posterior to the stimulation with F2Lipo-MP-LPS (TNF-α, RANTES (CCL-5), CXCL11 and BCL2-A1) whereas the M2 genes (MRC-1 and CCL13) were reduced. Similar observations have been made with unmodified LPS and thus suggest that both the liposomal formulation and the detoxification process do not alter the immunological properties of LPS, notably on macrophage polarization (57, 58). The impact of F2Lipo-MP-LPS on macrophages polarization must contribute to its potent antitumor effects as M1 macrophages are historically regarded as being anti-tumoral, while M2-polarized macrophages, commonly deemed tumor-associated macrophages (TAMs), are contributors to many pro-tumorigenic outcomes in cancer through angiogenic and lymphangiogenic regulation, immune suppression, hypoxia induction, tumor cell proliferation, and metastasis (59). In the recent reviews, Kashfi K et al. and Poltavets AS et al. suggested that M2 reprogramming toward a M1 phenotype is considered as an attractive new approach for cancer therapy (34, 60).

Our results thus showed that an innovative detoxification process, and a clinically approved liposomal formulation, can act synergistically to develop a systemically active and tolerable LPS-based TLR4 agonist retaining potent antitumor and adjuvant effects. This product could overcome the clinical limitations of current LPS derivatives restricted to local administration by inducing systemic activation of the immune system enabling to address tumors, not amenable to intratumoral injection, as well as disseminated tumors and metastatic cancers. Potential indications and combinations of systemic TLR4 agonist are likely to be very diverse as exemplified by our results in colorectal and lymphoma models, both as monotherapy and in combination with a therapeutic monoclonal antibody like rituximab. Pyrogenicity data in rabbits, a species considered to be as sensitive to LPS as humans, as well as toxicity data in dog, demonstrated that F2Lipo-MP-LPS presents a tolerance profile significantly better than Lipo-LPS in systemic administration, enabling to consider the administration of high doses. Taken together, our results are therefore very promising in order to consider evaluation of intravenously administered liposomal detoxified LPS in patients.

## 5 Conclusion

Our study provides strong preclinical data supporting the feasibility and safety of intravenous administration of Lipo-MP-LPS. These results strongly support the evaluation of this agent as a systemically active agent in patients with cancer.

## Data availability statement

The original contributions presented in the study are included in the article/**Supplementary Material**. Further inquiries can be directed to the corresponding author.

## Ethics statement

The animal study was reviewed and approved by This study was approved by the CECCAPP Animal Ethics committee. French Ministry of Higher Education and Research, France. Rodent experimental procedures were approved by the University of Lyon Animal Ethics committee and experiments in dogs were approved by the Institut Claude Bourgelat Animal Ethics committee.

## Author contributions

KC, CF, MD, AN, LJ, CP, JK, MC, and CD designed the experiments; KC, CF, MD, DM, PC, SB, EF, PN, DK, AE, CP, EP, E-LM, AN, and MC conducted the experiments; KC, MD, AN, JK, MC, and CD wrote the paper. All authors contributed to the article and approved the submitted version.
