# Supplementary material for: A systemically administered detoxified TLR4 agonist displays potent antitumor activity and an acceptable tolerance profile in preclinical models
Source: Front Immunol. 2023 May 8;14:1066402. doi: 10.3389/fimmu.2023.1066402 (PMC10200957; doi:10.3389/fimmu.2023.1066402)
Supplement: Supplementary file 1 [file DataSheet_1.pdf]

**Supplementary Figure 1**: Gating strategy used for flow cytometry analysis of the effect of Lipo-LPS on the production of reactive oxygen species (ROS) by granulocytes.

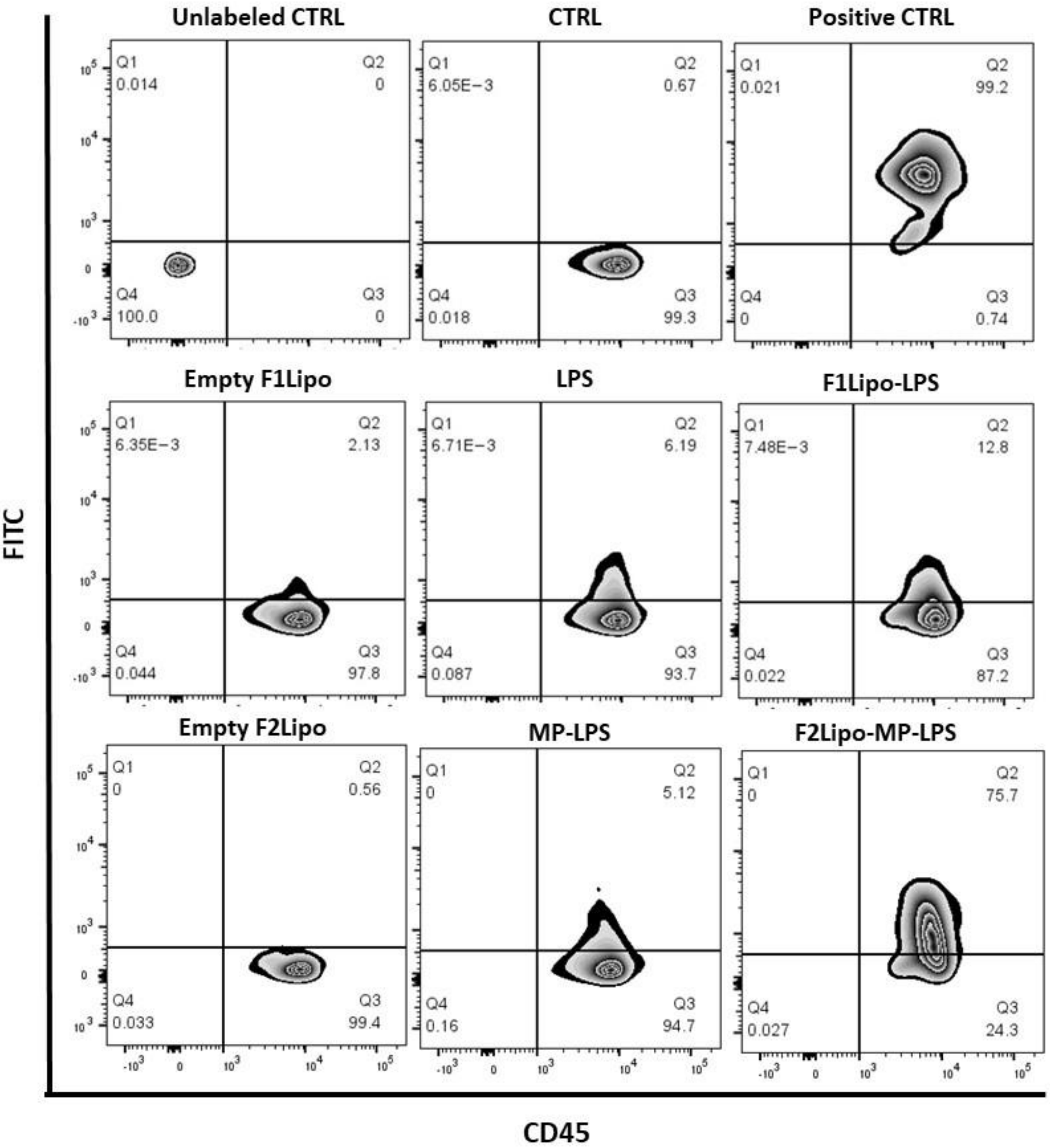

**Supplementary Figure 2**: **Gating strategies used in the study**. Representative gating strategy for immune cells after acquisition of MC38 tumor and spleens of mice untreated or treated with empty-F1Lipo or F1Lipo-LPS, gated for single live CD45+ cells. **(A)** Lymphoid subpopulation. **(B)** Myeloid subpopulation.

## Supplementary figure 2A

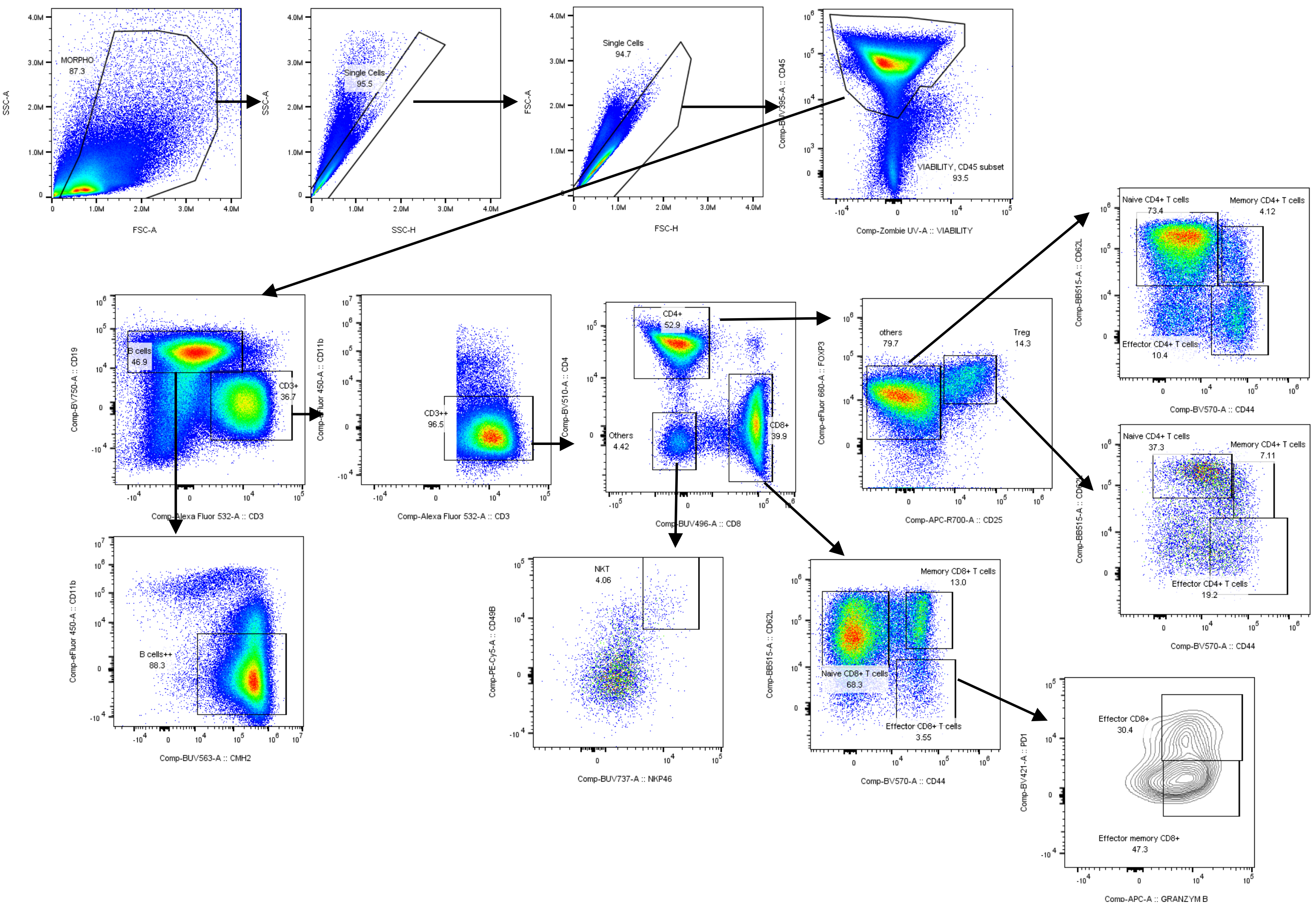

## Supplementary figure 2B

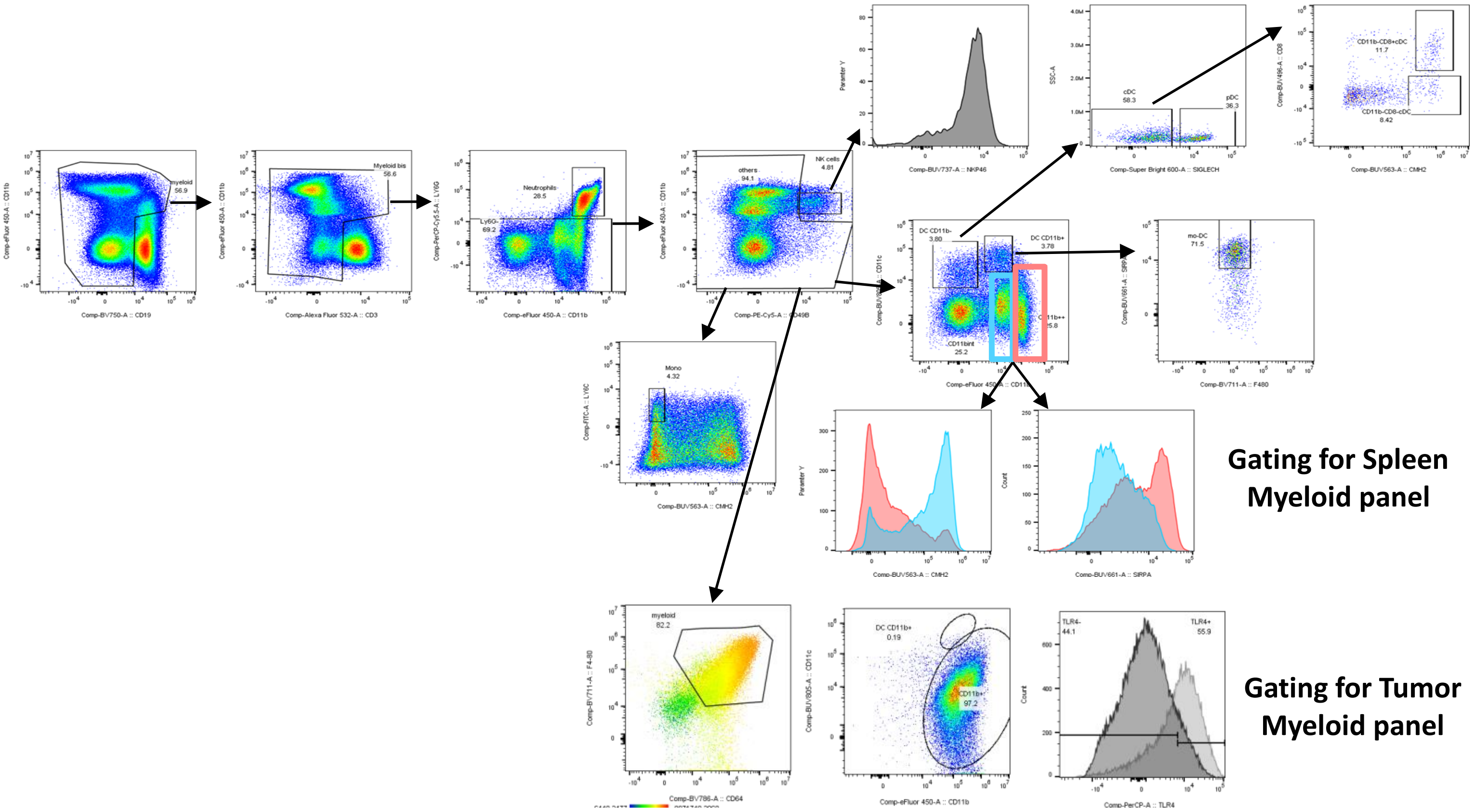

**Supplementary figure 3. Confocal microscopy analysis of fluorescently labeled liposomes. (A)** Confocal fluorescence image of a single F1Lipo-LPS liposome tagged on its lipid bilayer with Rhodamine B (F1Lipo-RhB-LPS); **(B)** intensity profile of image in (A); **(C)** confocal fluorescence image of a Rhodamine B-labeled liposome (red) showing the presence of encapsulated LPS-FITC (green) in the lipid bilayer.

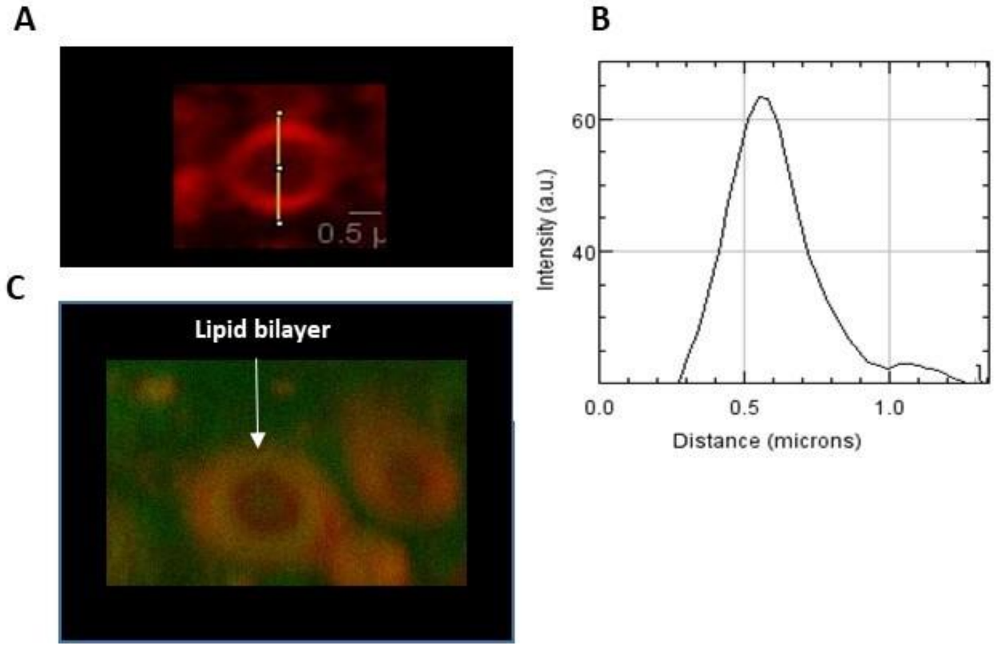

**Supplementary Figure 4: *In vitro* binding of liposomal formulations to fresh human leukocytes.** After blood collection in BD Vacutainers containing lithium heparin as an anticoagulant, 200 µl of normal human blood was distributed into 1.5 mL Eppendorf tubes containing emptyF1Lipo-NBD, F1Lipo-NBD-LPS**,** emptyF2Lipo-NBD and F2Lipo-NBD-MP-LPS and incubated for 3 h at 37°C with rotation. After incubation, red blood cells were removed using a lysis solution (BD Pharm Lyse, BD Biosciences). The remaining cells were incubated with human IgG for 10 min on ice to block unspecific binding and stained in 100 µL PBS for 30 min at 4°C with APC anti-human CD45 before being subjected to flow cytometry. The binding of leukocytes with DNB-liposomes (Ex 467 nm/Em 539 nm) was monitored by flow cytometry.

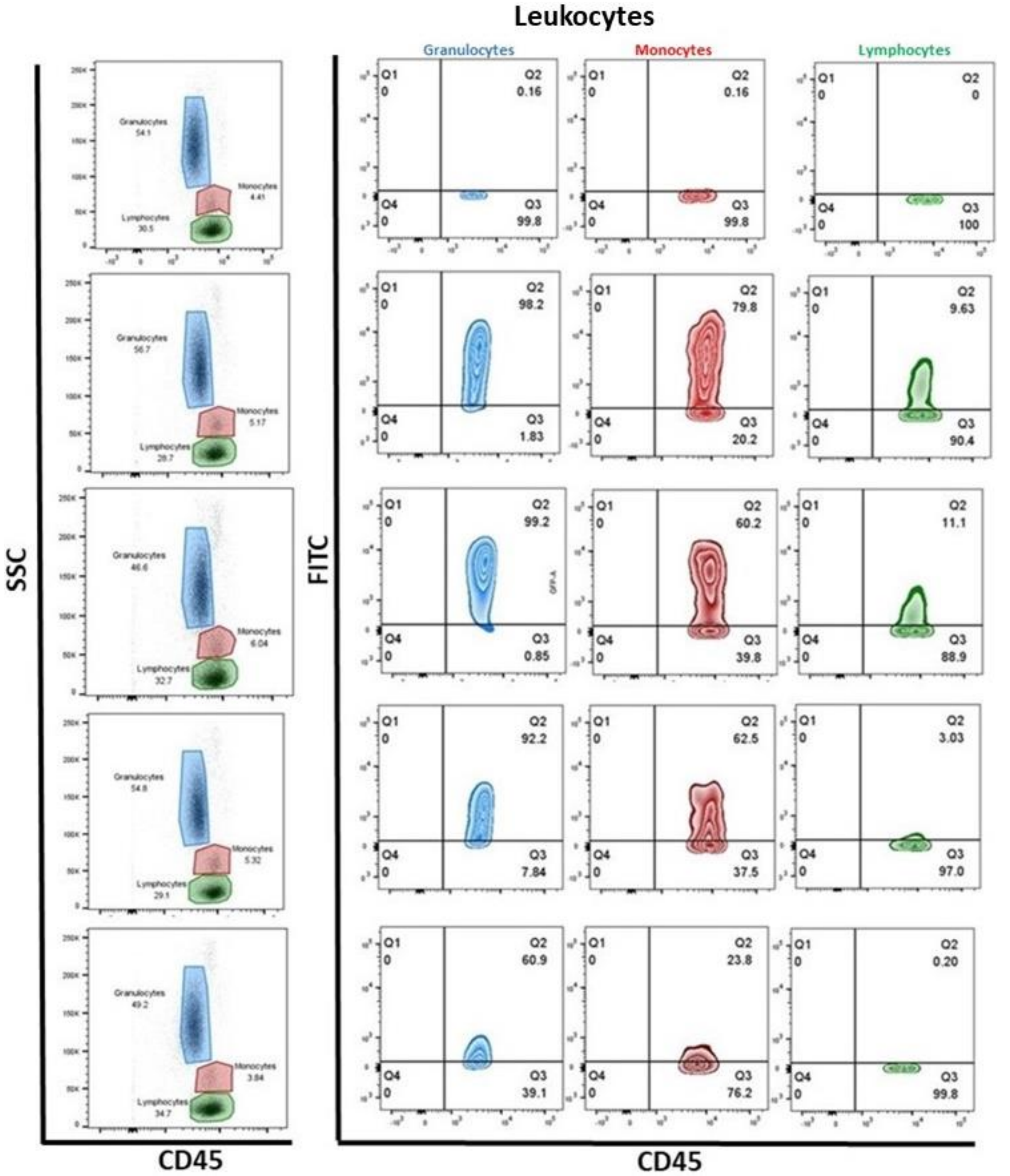

**Supplementary Figure 5: Binding of F1Lipo-LPS-FITC to fresh mice splenocyte leukocytes.** Spleen was collected in BALB/C mouse, after overnight culturing, splenocyte leukocytes were incubated 3 hours with or without F1Lipo-LPS-FITC. F1Lipo-LPS-FITC expression levels on CD4+ T (CD3+, CD4+) CD8+ T (CD3+, CD8+) CD19+ B (CD3- CD19+), monocytes (CD11b+, Ly6C++), macrophages (CD11b+, Ly6C-/+) and neutrophils (CD11b+, Ly6C+, Ly6G+) cells were measured by flow cytometry.

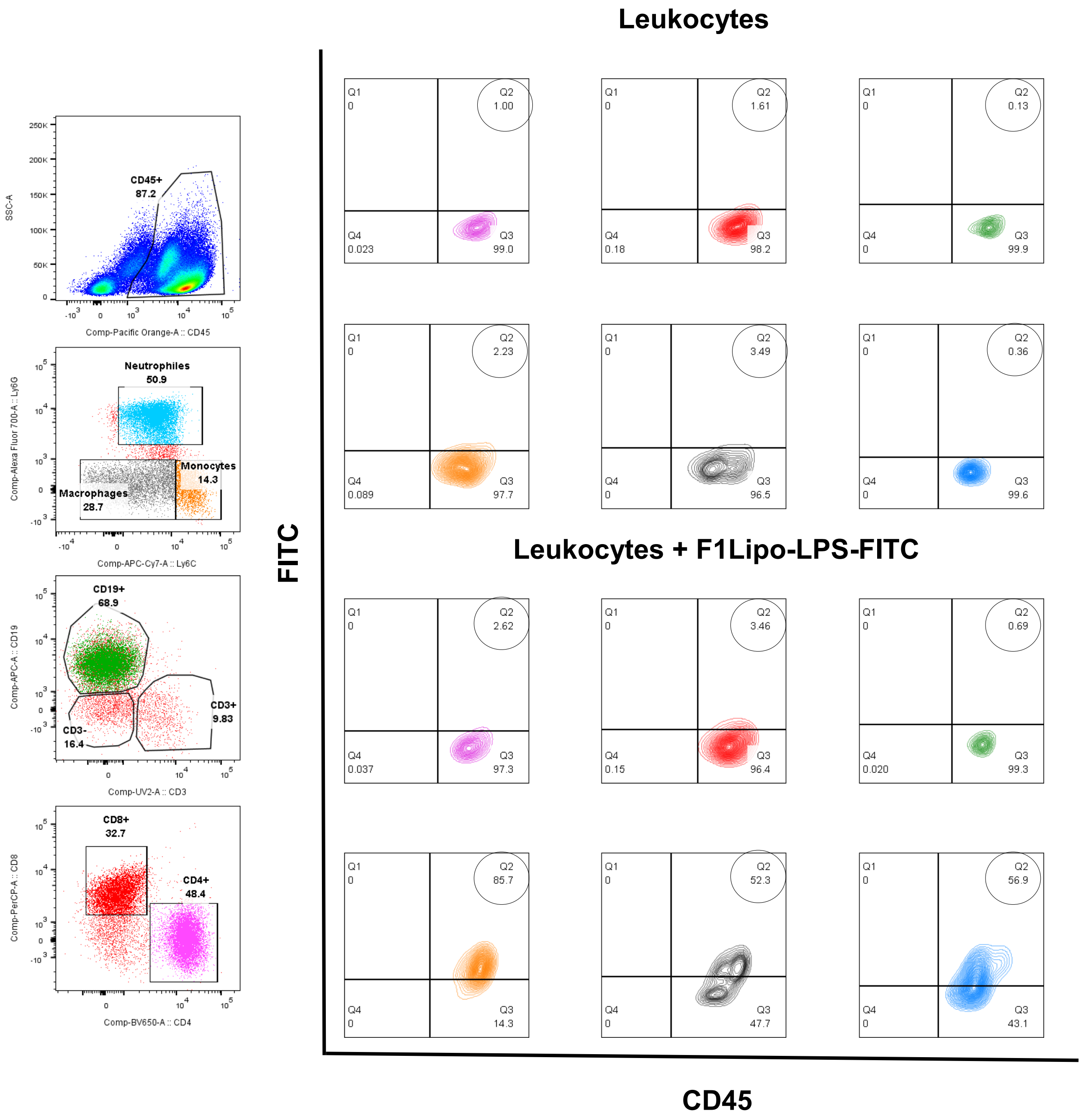

**Supplementary Figure 6: F2Lipo-MP-LPS localization**

C57BL/6 mice bearing established colorectal MC38 tumors were intravenously injected with $^{111}$In-labelled F2Lipo-MP-LPS. Distribution of $^{111}$In in tissues 24 hours post administration. (A) the total amount bound to liver was greater than that bound to spleen. (B)$^{111}$In-labelled F2Lipo-MP-LPS exhibits a similar binding on a per gram basis to spleen and live

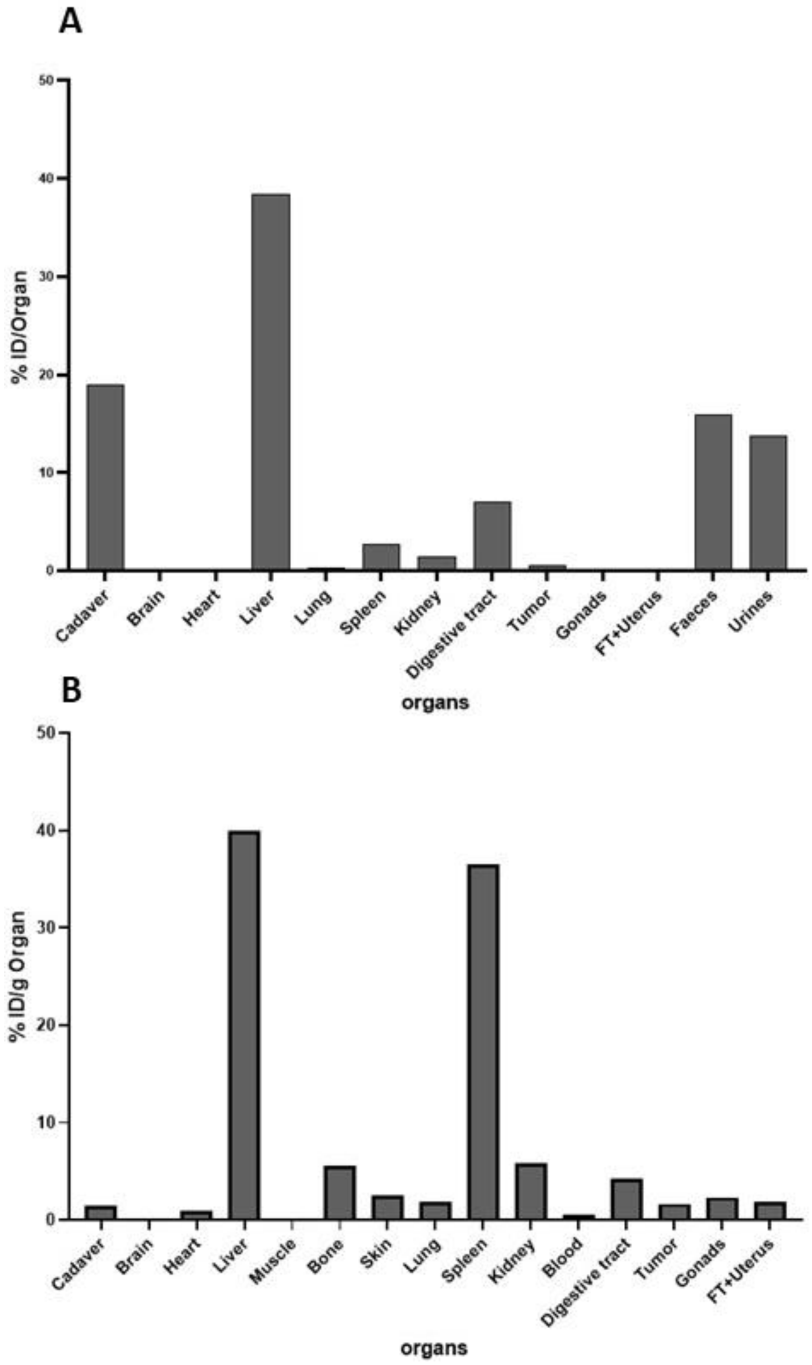

**Supplementary Figure 7: Details of immune subpopulations in mice after intravenous administration of F1Lipo-LPS treatment**. MC38 tumor cell lines were injected in C57Bl/6 mice, when tumors reached 100 $mm^3$, mice were randomized and treated with F1Empty Lipo or F1Lipo-LPS, immunophenotypic analyses (Cytek® Aurora) was performed in **(A)** tumors and **(B)** spleens. Flow cytometry analysis was performed 24h after the second weekly treatment administration. Subpopulation for CD8+ T, CD4+ T and Treg cells were analyzed for tumors and spleen samples. Histograms for each immune population were performed after normalization of the number of cells in 100,000 CD45+ viable events. Significant decreases and increases were assessed by a two-way ANOVA statistical test, with Bonferroni post-hoc test. n = 4 to 5 tumors per group, and n=3 to 5 spleens per group, for each *: p<0.05, ****: p<0.0001.

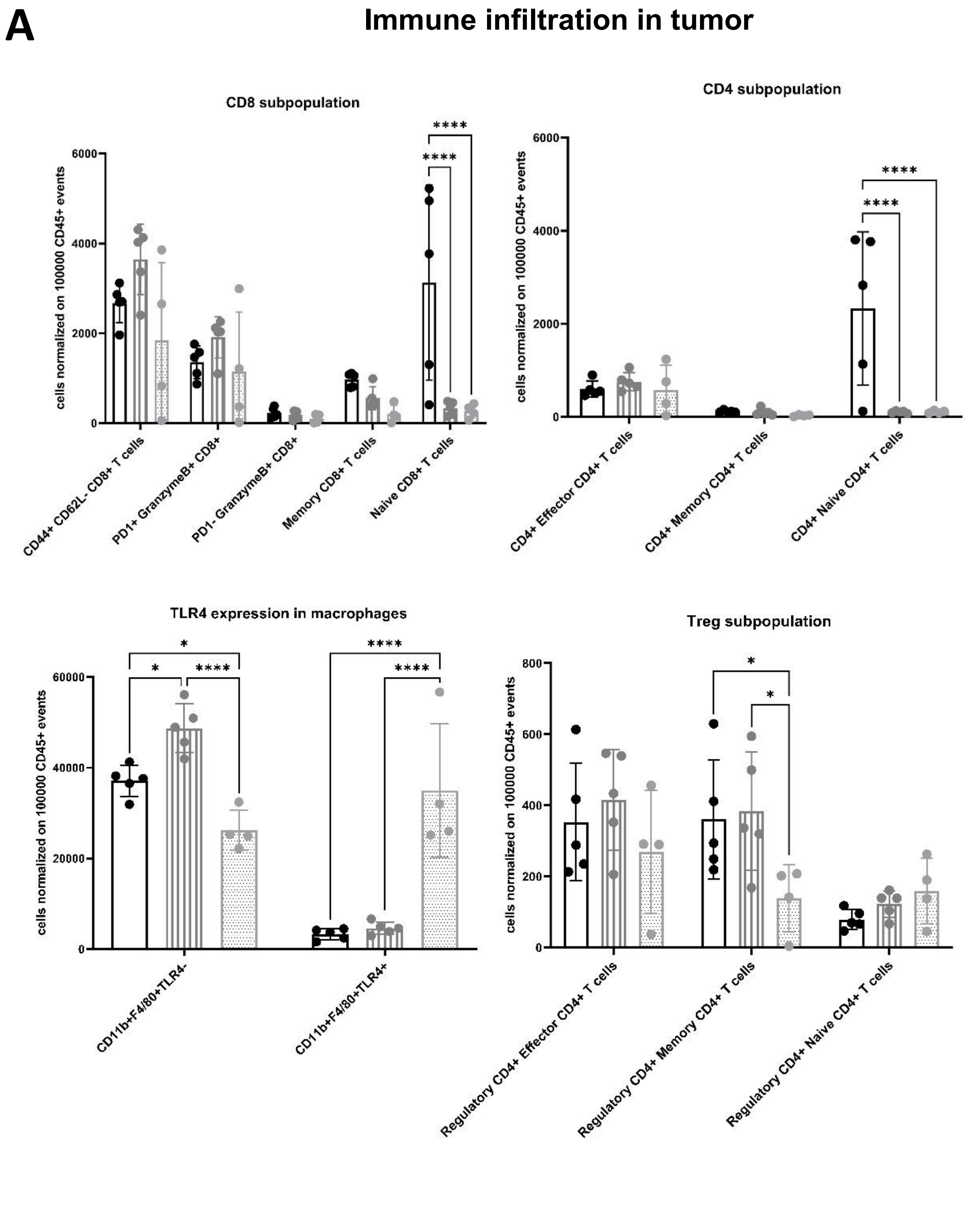

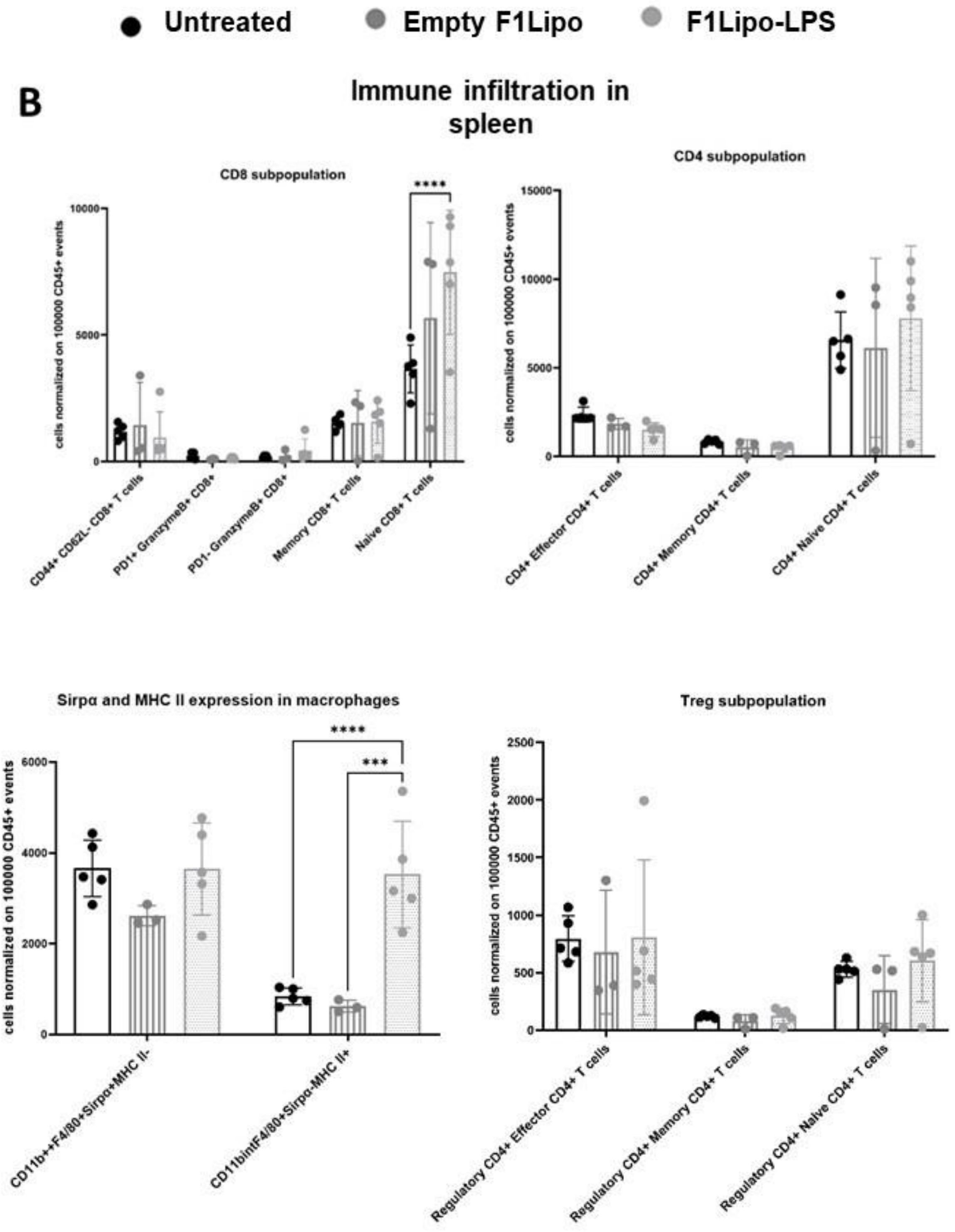
Untreated
Empty F1Lipo
F1Lipo-LPS
B
Immune infiltration in spleen
CD8 subpopulation
cells normalized on 100000 CD45+ events
****
CD44+ CD62L- CD8+ T cells
PD1+ GranzymeB+ CD8+
PD1- GranzymeB+ CD8+
Memory CD8+ T cells
Naive CD8+ T cells
CD4 subpopulation
CD4+ Effector CD4+ T cells
CD4+ Memory CD4+ T cells
CD4+ Naive CD4+ T cells
Sirpα and MHC II expression in macrophages
****
***
CD11b++F4/80+Sirpα+MHC II-
CD11bintF4/80+Sirpα-MHC II+
Treg subpopulation
Regulatory CD4+ Effector CD4+ T cells
Regulatory CD4+ Memory CD4+ T cells
Regulatory CD4+ Naive CD4+ T cells

**Supplementary Figure 8: Potentiation of the antitumor activity of F1Lipo-LPS in combination with GA101 antibody.** Granta tumor cell lines were injected in SCID mice subcutaneously, when tumors reached 100 mm$^3$, mice were randomized and treated with Empty F1Lipo, F1Lipo-LPS, or GA101 alone or in combination. Data shown are mean tumor volume values and error bars are ± SEM n=5 mice/group.

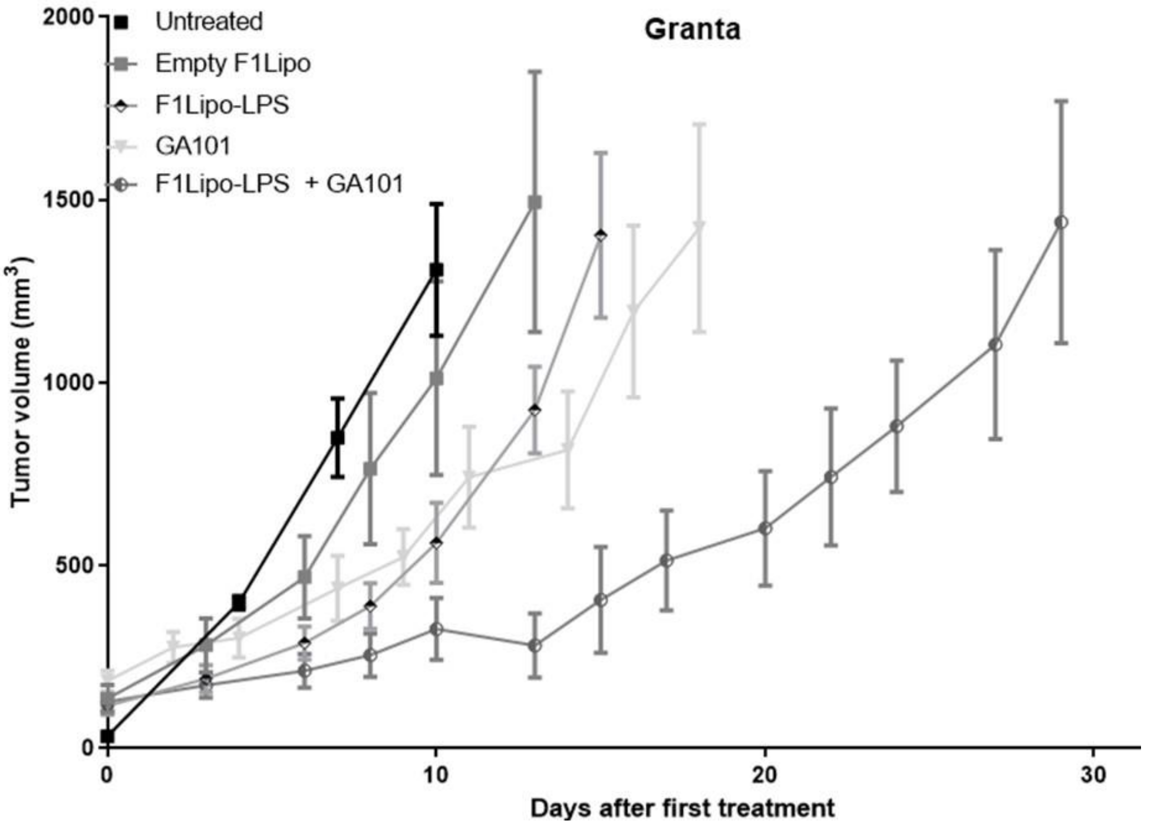

**Supplementary figure 9**: **Antitumor activity of F1Lipo-LPS in the orthotopic osteosarcoma model K7M2 and the syngeneic NHL model A20. (A)** K7M2 tumor cells lines were injected in the femur of BALB/C, then 24h after injection mice were treated with F1Lipo-LPS. **(B)** A20 tumor cell lines were injected in BALB/C subcutaneously, when tumors reached 100 $mm^3$, mice were randomized and treated with F1Lipo-LPS. Data shown are mean tumor volume values and error bars are ± SEM n=5 mice/group, **: p>0.01, using Mann Whitney t-test for A20 experiment. For K7M2 experiment, Data shown are survival curves n=6 mice/group, *: p>0,05, using Log-rank (Mantel-Cox) test.

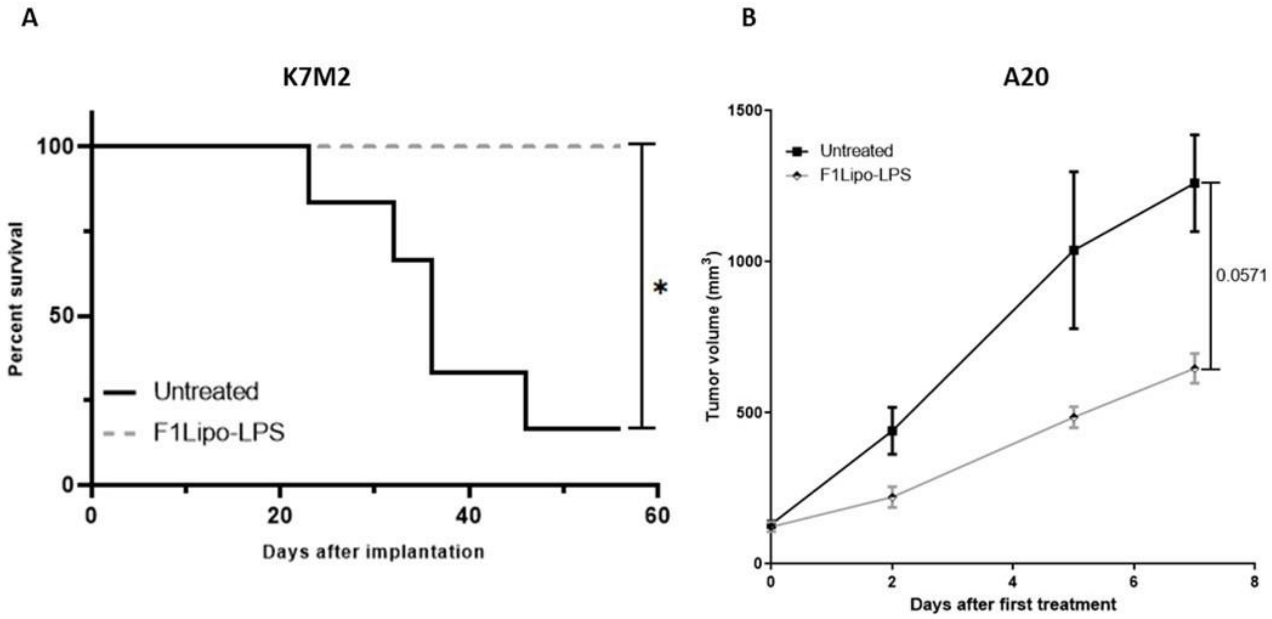

**Supplementary Figure 10: Potentiation of the antitumor activity of F1Lipo-LPS in combination with anti PD-1 antibodies.** MC38 tumor cell lines were injected in C57B/6 mice subcutaneously. When tumors reached 100 $mm^3$, mice were randomized and treated with Empty F1Lipo, F1Lipo-LPS, or anti PD-1 antibodies alone or in combination. Data shown are mean tumor volume values and error bars are ± SEM n=5 mice/group, **: p>0.01, using Mann Whitney t-test.

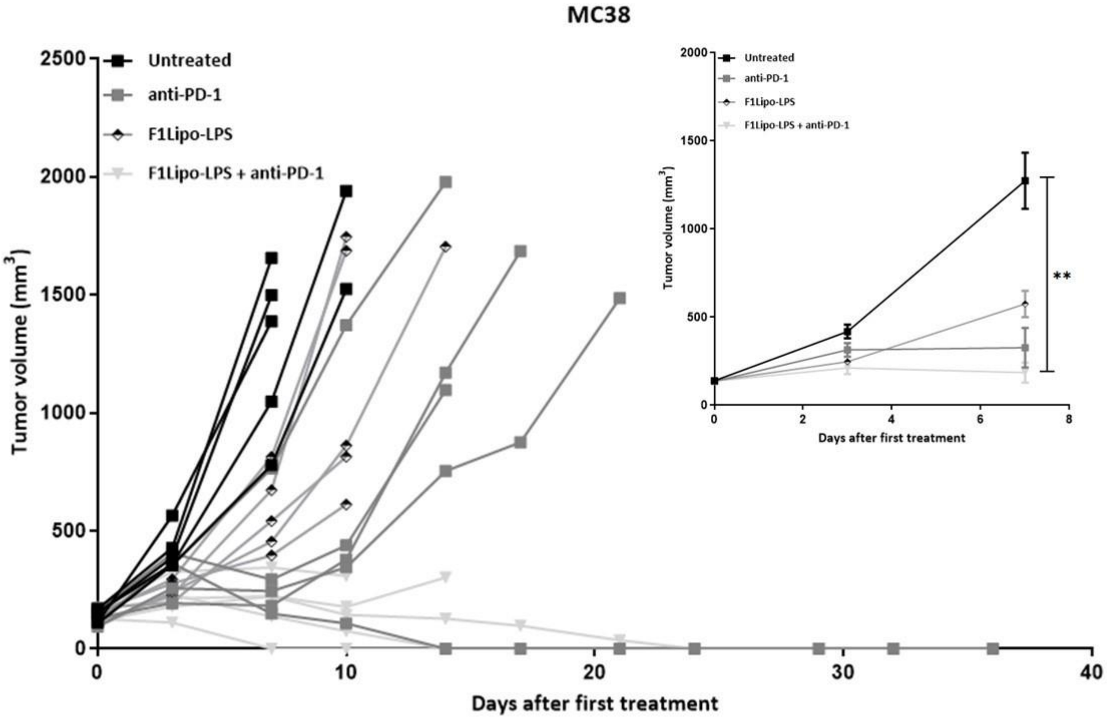

**Supplementary Figure 11**. **Rabbit pyrogenicity assay**. Pyrogenicity in rabbits following administration of 0.75 ng/kg of LPS, 175 ng/kg of free MP-LPS versus 1750 ng/kg of F2Lipo-MP-LPS. A compound is considered pyrogen free if the summed response of the differences between the highest temperature 3 hours post-injection and baseline temperature for three rabbits does not exceed 1.15°C, and fails if the summed response exceeds 2.65°C.

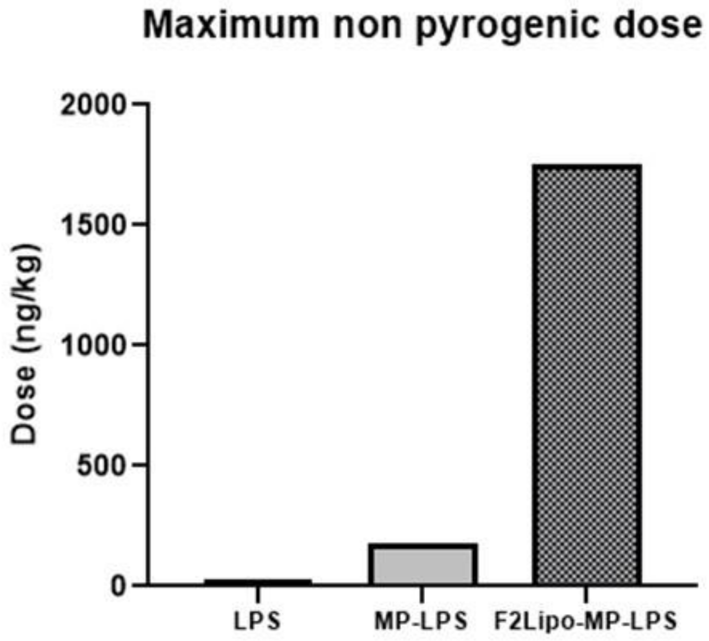

**Supplementary Table 1: List of primers used to analyze cytokines induction.**

| Name | Sequence |
|---|---|
| IL1β For | TACCTGTCCTGCGTGTTGAA |
| IL1β Rev | TCTTTGGGTAATTTTTGGGATCT |
| TNFalpha For | CAGCCTCTTCTCCTTCCTGAT |
| TNFalpha Rev | GCCAGAGGGCTGATTAGAGA |
| MRC1 For | CTGAATTGTACTGGTCTGTCCT |
| MRC1 Rev | GCCAGGCTAGAAAACAAGCAA |
| Rantes For | ACAGGTA CCATGAAGGTCTC |
| Rantes Rev | TCCTAGCTCATCTCCAAAGA |
| CXCL1 For | GCGCCCAAACCGAAGTCATA |
| CXCL1 rev | ATGGGGGATGCAGGATTGAG |
| IP-10 For | GGTGAGAAGAGATGTCTGAATCC |
| IP-10 Rev | GTCCATCCTTGGAAGCACTGCA |
| IFIT1 For | GCCTTGCTGAAGTGTGGAGGAA |
| IFIT1 Rev | ATCCAGGCGATAGGCAGAGATC |
| BCL2-A1 For | CAGGAGAATGGATAAGGCAAA |
| BCL2-A1 Rev | CCAGCCAGATTTAGGTTCAAA |
| CCL13 For | AGCCAGATGCACTCAACGTC |
| CCL13 Rev | TCTCCTTGCCCAGTTTGGTT |
| IFNγ For | TTTTGAAGAATTGGAAAGAGGA |
| IFNγ Ror | CACTTGGATGAGTTCATGTATTG |
| COX-2 For | CGGTGAAACTCTGGCTAGACAG |
| COX-2 Rev | GCAAACCGTAGATGCTCAGGGA |
| IFNβ For | CTCCTGTTGTGCTTCTCCACT |
| IFNβ Rev | GGCAGTATTCAAGCCTCCCA |
| CXCL-11 For | AGTGTGAAGGGCATGGCTA |
| CXCL-11 Rev | CTGCAGGGTCGACACATCT |

**Supplementary Table 2: List of antibodies used for flow cytometry analysis of the tumor immune microenvironment.**

| Antigen | Clone | Labelling | Fluorochrome | Supplier | Cat number | RRID |
|---|---|---|---|---|---|---|
| CD25 | PC61 | EXTRA | APC-R700 | BD | 565134 | AB_2744344 |
| CD45 | 30-F11 | EXTRA | BUV395 | BD | 564279 | AB_2651134 |
| CD8 | 53,6,7 | EXTRA | BUV496 | BD | 750024 | AB_2874242 |
| CMH2 | M5/114,15,2 | EXTRA | BUV563 | BD | 748846 | AB_2873249 |
| SIRPa | P84 | EXTRA | BUV661 | BD | 741593 | AB_2871002 |
| NKp46 | 29A1,4 | EXTRA | BUV737 | BD | 612805 | AB_2870131 |
| CD11c | N418 | EXTRA | BUV805 | BD | 749038 | AB_2873432 |
| CD38 | HB7 | EXTRA | BV650 | BD | 740489 | AB_2740212 |
| CD19 | 1D3 | EXTRA | BV750 | BD | 747332 | AB_2872036 |
| CD64 | X54-5/7,1 | EXTRA | BV786 | BD | 741024 | AB_2740644 |
| Ly6C | AL-21 | EXTRA | FITC | BD | 561085 | AB_10584332 |
| CD62 L | MEL-14 | EXTRA | BB515 | BD | 565261 | AB_2739138 |
| CD206 | C068C2 | INTRA | PE-Dazzle594 | Biolegend | 141732 | AB_2565932 |
| PD-1 | RMP1-30 | EXTRA | BV421 | Biolegend | 109121 | AB_2687080 |
| CD4 | RM4-5 | EXTRA | BV510 | Biolegend | 100559 | AB_2562608 |
| CD44 | IM7 | EXTRA | BV570 | Biolegend | 103037 | AB_10900641 |
| F4/80 | BM8 | INTRA | BV711 | Biolegend | 123147 | AB_2564588 |
| T-Bet | 4B10 | INTRA | PE | Biolegend | 644810 | AB_2200542 |
| Ly6G | 1A8 | EXTRA | PercP-Cy5.5 | Biolegend | 127616 | AB_1877271 |
| Viability UV Zombie | | EXTRA | | Biolegend | 423108 | |
| TLR4 | MTS510 | EXTRA | PercP | Novus Biologicals | NBP2-24865PCP | |
| CD3 | 17A2 | EXTRA | AF532 | Thermo | 58-0032-82 | AB_11217479 |
| Granzyme B | GB12 | INTRA | APC | Thermo | MHGB05 | AB_10373420 |
| CD11b | M1/70 | EXTRA | eF450 | Thermo | 48-0112-82 | AB_1582236 |
| FoxP3 | FJK-16s | INTRA | eF660 | Thermo | 50-5773-82 | AB_11218868 |
| CD49b | DX5 | EXTRA | PE-Cy5 | Thermo | 15-5971-82 | AB_2573070 |
| CD24 | M1/69 | EXTRA | PercP-eF710 | Thermo | 46-0242-82 | AB_1834425 |
| SiglecH | eBio440c | EXTRA | SB600 | Thermo | 63-0333-82 | AB_2784853 |
| PD-L1 | MIH5 | EXTRA | PE-CY7 | Thermo | 25-5982-82 | AB_2573509 |
| CD107a+ | 1D4B | EXTRA | APC-e780 | Thermo | 47-1071-82 | AB_2848363 |
